# High-speed photoacoustic and ultrasonic computed tomography of the breast tumor for early diagnosis with enhanced accuracy

**DOI:** 10.1126/sciadv.adz2046

**Published:** 2025-10-08

**Authors:** Keer Huang, Peifen Fu, Hengyue Zhu, Jingyi Feng, Liang Zhang, Baohua Wang, Yuexin Lu, Di Zhang, Minya Yao, Luyan Chen, Yue Ying, Jiali Chen, Xiaolong Li, Yang Wu, Wei Xiong, Jun Li, Yaping Wu, Jing Sun, Hong Zhang, Li Lin

**Affiliations:** ^1^ The First Affiliated Hospital, Zhejiang University School of Medicine, Hangzhou 310003, China.; ^2^ College of Biomedical Engineering and Instrument Science, Zhejiang University, Hangzhou 310027, China.; ^3^ Nanhu Brain-computer Interface Institute, Hangzhou 311121, China.

## Abstract

We have developed a high-speed dual-modal imaging system (HDMI), designed to concurrently reveal anatomical and hematogenous details of the human breast within seconds. Through innovative system design and technical advancements, HDMI integrates large-view photoacoustic and ultrasonic computed tomography with standardized scanning and batch data processing for computer-aided diagnosis. It achieves dual-modal imaging at a 10-hertz frame rate and completes a whole-breast scan in 12 seconds, providing penetration up to 5 centimeters in vivo. In a clinical study involving 170 patients with 186 breast tumors, we developed a diagnostic model leveraging combined photoacoustic and ultrasound features. In a triple-blinded comparison using pathological diagnosis as the ground truth, HDMI significantly improved diagnostic specificity from 22.5 to 75.0% compared to clinical ultrasonography. This technology shows strong potential for early breast tumor diagnosis, offering enhanced accuracy without the need for ionizing radiation, exogenous contrast agents, pain, invasiveness, operator dependence, or extended examination times.

## Teaser

A dual-modal photoacoustic-ultrasonic tomography system enables rapid, accurate breast tumor diagnosis with improved specificity.

## INTRODUCTION

Breast cancer is the leading cause of cancer-related mortality among women globally, with a notable rise in invasive cases over the past decade (*1*). Screening, diagnosis, and treatment are the key clinical sessions aimed at the detection, classification, and eradication of lesions. Early diagnosis, usually performed upon lesion detection, plays a critical role because the grading outcomes of the tumor directly determine clinical pathways, ranging from vigilant observation to core biopsy, fine-needle aspiration, advanced imaging modalities such as contrast-enhanced magnetic resonance imaging (CE-MRI) (*2*, *3*), or vacuum-assisted excision with minimal invasiveness. Accordingly, early diagnosis of breast tumors typically resorts to noninvasive imaging techniques characterized by high speed, precision, standardized operation, minimal ionizing radiation exposure, and the absence of contrast agent injection. In response to these prerequisites, x-ray mammography and ultrasonography have emerged as the primary modalities for this purpose. Although each modality has distinct advantages, mammography exhibits lower sensitivity in women with radiographically dense breasts (*4*, *5*), while ultrasonography suffers from reduced specificity due to the lack of physiological information (*6*). Consequently, in current clinical settings, a substantial proportion of patients referred to the biopsy department ultimately receive diagnoses of benign masses (57 to 86%) (*7*, *8*), notably increasing the workload and burden for surgeons, pathologists, and patients.

Complementing conventional imaging modalities, photoacoustic (PA) (or optoacoustic) computed tomography (PACT) offers a hybrid approach for mapping optical absorption properties beyond the optical diffusion limit while maintaining high spatiotemporal resolution (*9*). PACT typically involves the safe illumination of biological tissues with pulsed laser light, inducing local temperature increases in optical chromophores and subsequent pressure rises that propagate through soft tissues as ultrasonic waves (i.e., PA signal). Ultrasonic transducers positioned around the biological tissue detect the time-resolved PA signals, enabling the reconstruction of the spatial distribution of specific optical chromophores with acoustic resolution (*10*). Because the ultrasonic arrays and data acquisition (DAQ) modules used in PACT are compatible with ultrasonography, coregistered ultrasound images can be acquired with slight modifications to a PACT system by adding ultrasound transmission circuits. This combination enhances current breast imaging methodologies by providing additional physiological insights without the need for exogenous contrast agent injection.

From a physiological perspective, localized hypoxia initially arises in breast tumors due to uncontrolled cell growth, inducing the secretion of epidermal growth factor and subsequent irregular proliferation of blood vessels—a process called angiogenesis, which is recognized as a hallmark of breast cancer (*11*, *12*). Clinical evidence underscores that angiogenesis typically begins as early as the pathogenesis of simple breast hyperplasia (*13*, *14*) or the breast carcinoma in situ stage (*15*, *16*), highlighting its central role in cancer progression, invasion, and metastasis (*17*, *18*). In addition to increased quantity and irregular morphology (*19*, *20*), newly formed blood vessels often exhibit enhanced permeability, facilitating tumor delineation via CE-MRI through the extravasation of gadolinium. Because oxyhemoglobin (HbO_2_) and deoxyhemoglobin predominantly govern optical absorption in the visible and near-infrared spectral regimes, both angiogenesis and blood oxygen saturation (sO_2_) serve as intrinsic sources of PA imaging contrast (*21*). Accordingly, PACT holds promise for providing accurate early diagnosis through this contrast mechanism, potentially reducing the number of patients subjected to unnecessary biopsies. Moreover, the high imaging speed, safe operation, and provision of physiological information make PACT suitable for serial imaging to monitor and assess cancer responses to treatment (*21*, *22*).

From a technical perspective, breast imaging with clear tumor visualization represents one of the most challenging applications of PACT, owing to the demands for deep tissue penetration, large field of view (FOV), fast imaging speed, and high system reliability. Recently, several research groups have extended this technology from small animal to human breast imaging, developing PACT systems that use various acoustic detection geometries, including cylindrical (*23*), hemispherical (*24*–*28*), linear (*29*–*33*), and planar (*34*, *35*) arrays. These systems have facilitated substantial progress in breast cancer screening, diagnosis, and therapeutic assessment. For example, cylindrical detection geometry has been used in the single-breath-hold PACT system, which has been demonstrated for breast cancer detection (*23*), neoadjuvant chemotherapy assessment (*22*), and tumor diagnosis in a cohort of 39 patients (*36*). Hemispherical detection matrix has been implemented in the photoacoustic and ultrasound transmission tomographic system, which features dual-modality integration and a penetration depth of up to 48 mm in human breasts (*37*). Despite these advances, most clinical trials using cylindrical and hemispherical arrays have yet to produce statistically significant diagnostic outcomes (*22*–*28*, *36*, *37*), and the reproducibility of their clinical performance remains insufficiently evaluated. Nevertheless, these configurations typically offer superior image quality and a wider FOV compared to PACT systems based on linear arrays, which are more commonly adopted for dual-modal PACT and B-mode ultrasound imaging. For instance, Seno Medical Instruments (*38*–*40*) modified a commercial ultrasound platform by integrating optical modules and imaged breast masses initially classified as Breast Imaging Reporting and Data System (BI-RADS) 3, 4, or 5. The addition of PACT enhanced the diagnostic specificity of stand-alone ultrasonography by 14.9% (*P* < 0.0001) (*39*, *41*). Similar configurations have been deployed in other breast diagnostic studies, showing differences in hemoglobin concentration and sO_2_ between benign and malignant tumors (*42*).

Although it maintains most functions of an ultrasound system, the “commercial ultrasound plus laser” approach has three crucial drawbacks: (i) Handheld PACT still exhibits limitations in operator-dependent performance, leading to variability in diagnostic outcomes. (ii) PACT, relying solely on acoustic signal detection without beam steering emission, suffers from restricted view angles in linear ultrasonic arrays, notably compromising image quality. For instance, linear array–based PACT struggles to detect blood vessels oriented perpendicular to the array (*43*). (iii) Commercial ultrasonography typically uses probes and electronics that are suboptimal for PACT. PA signals from deep tissues tend to be weaker than those in B-mode ultrasound, necessitating high-end PACT systems with detection-optimized ultrasonic transducers closely connected to low-noise preamplification circuits (*23*, *24*).

Here, we present progress in breast PACT, addressing previously identified technical constraints and demonstrating a statistically significant improvement in early breast tumor diagnosis. Our breast imager—high-speed dual-modal imaging system (HDMI)—integrates both PACT and ultrasonic reflection-mode computed tomography (URCT) modalities. It stands out as the first PACT system to simultaneously fulfill the following conditions: (i) Combination of dark-field optical illumination with large-view acoustic detection allows HDMI to clearly visualize small blood vessels up to 5 cm beneath the breast skin surface with minimal artifacts. (ii) Duplex use of a half-ring ultrasonic array and DAQ modules enables the acquisition of large-aperture PACT and URCT images in parallel, facilitating their fusion in a unified image coordinate system. (iii) HDMI can perform standardized whole-breast scanning within 12 s or real-time dual-mode image reconstructions of arbitrary breast cross sections. (iv) Enhanced reproducibility in breast shape and positioning ensures consistent image quality across scans and participants through batch data processing. (v) Comprehensive analysis of PACT and URCT image features from batch-processed images enables accurate computer-aided diagnosis of breast tumors. Equipped with HDMI, we conducted clinical trials involving PACT and URCT scans of 170 patients with 186 breast tumors, categorizing them as either benign or malignant, without prior access to clinical diagnosis or histopathology findings. We demonstrated improvements in early diagnostic specificity by comparing HDMI’s results with those of clinical ultrasound performed on the same cohort of patients.

## RESULTS

### High-speed dual-modal imaging system

**Fig. 1.**
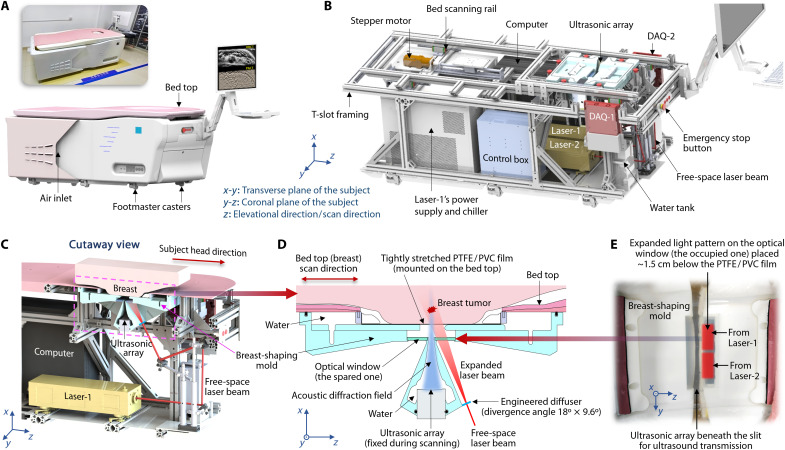
Representations of the HDMI system (preproduction prototype). (**A**) Photograph and perspective view of the preproduction prototype on wheels. (**B**) Perspective view of the preproduction prototype with the bed top, system enclosure, and some cooling fans removed. (**C**) Cutaway view of the front half of the system showing the relative positions of the compressed breast, breast-shaping mold, ultrasonic array, and laser beam. (**D**) Cross-up view of the pink dashed box in (C), showing the breast-shaping mold compressing the breast against the chest wall while also serving as a water tank for acoustic coupling. Laser beams are directed through one of the optical windows toward the breast. Ultrasonic signals can transmit through the slit between the two optical windows attached to the breast-shaping mold. (**E**) Photograph of the breast-shaping mold viewed from the top. Laser beams from two identical neodymium-doped yttrium aluminum garnet lasers are expanded by two engineered diffusers, creating two rectangular beam patterns on the optical window.

Diverging from previously published PACT systems, HDMI introduces a design incorporating multiple innovative configurations. As illustrated in Fig. 1 and fig. S1, all HDMI components and modules are installed beneath a scan platform. The setup involves a prone human participant positioned on the bed, with the breast to be imaged isolated from the imaging system by a tightly stretched 0.05-mm-thick polytetrafluoroethylene (PTFE) or polyvinyl chloride (PVC) film mounted on the bed board (see Materials and Methods). Both films introduce ~5% ultrasound loss during one-way transmission (fig. S2), while exhibiting negligible optical loss at 1064 nm. Beneath the stretched film, a breast-shaping mold supports and painlessly compresses the breast against the chest wall, ensuring consistent shaping for relatively large breasts (i.e., ≥B cups) (see Materials and Methods). This mold features a slit aligned with the ultrasonic array in the middle for ultrasound transmission and two transparent windows adjacent to the slit for optical illumination (Fig. 1, D and E).

The cylindrically focused half-ring ultrasonic array is vertically mounted beneath the mold, providing efficient acoustic detection and emission within breast cross sections on the transverse plane. Parallel laser beams emitted from two neodymium-doped yttrium aluminum garnet lasers are expanded by two engineered diffusers and directed toward the breast (fig. S1A). Because of space limitations and safety concerns (see Materials and Methods), both laser beams are aligned through a single optical window (Fig. 1, D and E). We innovatively use dark-field illumination in HDMI to minimize artifacts from strong surface PA signals (*44*) and reduce signal variations along depth, better fitting the dynamic range of DAQ systems (see Materials and Methods). By directing most of the light energy onto the skin surface outside the acoustically defined imaging plane, HDMI detects fewer PA signals originating from superficial tissues. In addition, a more uniform optical energy distribution can be achieved across the imaging plane by directing the laser beam obliquely to the skin surface (fig. S3).

**Fig. 2.**
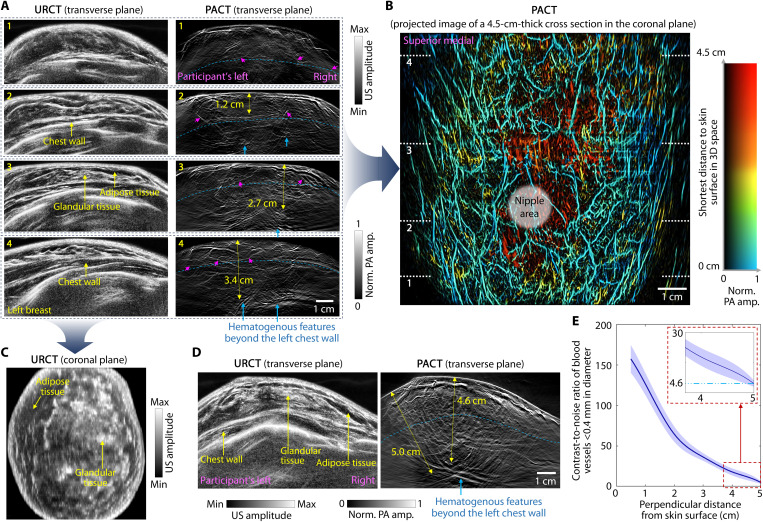
HDMI of left breasts in female volunteers. (**A**) Real-time URCT (left) and PACT (right) images of the same breast cross sections on the transverse plane, visualizing adipose tissue, glandular tissue, and chest wall structures in URCT images and blood vessels down to an apparent diameter of 0.2 mm in PACT images. Chest walls are indicated by blue dashed lines, with diving vessels from the chest wall to the breast tissue marked by magenta arrows. Hematogenous features beyond the chest wall are labeled with blue arrows. US, ultrasound. (**B**) Maximum amplitude projection (MAP) image of the whole-breast vasculature with color-encoded depths. Vasculature colors indicate the shortest distance of each voxel to the skin surface in 3D space. The white dashed lines indicate the positions of the transverse cross sections shown in (A). The light-colored nipple is not visible in the MAP image but can be identified in the transverse cross-sectional images. The entire scan was completed in a single breath hold of 12 s. (**C**) URCT image of the same breast, showing a 1-mm-thick cross section in the coronal plane. (**D**) Dual-mode image examples from another participant, revealing blood vessels as deep as 5 cm from the skin surface. In PACT images, we identify blood vessels based on their tubular structures with either branching or sinuous morphologies. (**E**) Contrast-to-noise ratio (CNR) of blood vessels with an apparent diameter of <0.4 mm measured at different depths from the skin surface. CNRs were quantified by selecting blood vessels with bifurcation features as highlighted in (A) and (D).

During each breast scan, the bed board moves linearly along the *z* axis (Fig. 1, C and D), perpendicular to the transverse imaging plane (*x*-*y*), covering a range of 85 to 120 mm in length to encompass the majority of the breast tissue. The two synchronized lasers emit pulsed light at a repetition rate of 10 Hz, generating cross-sectional PACT images with a depth of 50 mm and a width of 120 mm through real-time reconstruction (see Materials and Methods). Within the time interval between the two adjacent PACT frames (~99.8 ms), 30 evenly distributed transducer elements in the array sequentially emit ultrasound pulses with a large in-plane diffraction angle (38° to 48°) that propagate toward the breast within the cross-sectional area (fig. S4). The reflected ultrasound signals are detected by the remaining 480 elements (from 512 elements with 30 emitters and 2 reserved elements), facilitating large-view URCT (see Materials and Methods). Consequently, real-time PACT and URCT of breast cross sections are alternately conducted during the scan process (movie S1), enabling the display of whole-breast PACT and URCT images with spatial alignment (Fig. 2, A to D). Each cross-sectional PACT and URCT image can be acquired within 160 μs and 93 ms, respectively, allowing HDMI to provide two-dimensional (2D) images with negligible motion artifacts.

The HDMI design prioritizes real-time dual-modal 2D imaging to ensure clear visualization of tumor-associated angiogenesis, which typically consists of small and tortuous blood vessels. Even during a single-breath-hold scan, soft breast tissue is prone to motion caused by heartbeat perturbations and unconscious movements, often at scales comparable to or larger than those of small blood vessels (movie S2). Given that a PACT system capable of real-time volumetric imaging with a large FOV would require a substantial number of transducer elements, preamplifier circuits, and DAQ channels, we chose to prioritize real-time 2D imaging to ensure clear and reliable visualization of breast tumors. All cross-sectional PACT images are reconstructed using a unipolar weighted delay-and-sum (DAS) method (see Materials and Methods) (*45*, *46*), reducing visible artifacts typically associated with the limited FOV in bipolar images (figs. S5 and S6). Breast tissue–mimicking phantom studies were conducted to validate the penetration depth (fig. S7) and to investigate the impact of acoustic heterogeneity in breast tissue on PACT image reconstruction using a single speed of sound (fig. S8).

We initially developed a demonstration prototype (fig. S1B) and imaged 5 healthy volunteers and 12 patients with 12 breast masses to assess its performance and refine the design. In this prototype, we evaluated two ultrasonic arrays with identical dimensions and configurations but different center frequencies (2.25 and 3 MHz) for breast imaging. Because of electromagnetic interference affecting half of the elements in the 2.25-MHz array—likely resulting from inadequate grounding during its fabrication—we opted to use the 3-MHz array in the preproduction prototype (Fig. 1 and fig. S1C), which acquired all the image data presented in the manuscript. This configuration provided clear PACT and URCT images with penetration beyond the chest wall (Fig. 2, fig. S9, and movie S2), achieving a spatial resolution of 0.20 mm in the transverse plane and 2.3 mm along the scanning direction (fig. S10). We further developed a machine learning model (see Materials and Methods) to enhance the sharpness of image features in the scanning direction (fig. S11). Before systematic imaging and data batch processing, we demonstrated the reproducibility of HDMI in providing consistent-quality PACT/URCT images in identical or similar breast cross sections by imaging human participants multiple times (fig. S12).

### HDMI of patients with breast tumors

Of 158 consented patients (170 total, with 12 excluded) with 174 breast masses imaged by the preproduction prototype, 5 patients (with seven masses) underwent HDMI for system optimization, program testing, and operator training. An additional five patients (with five masses) were excluded because of incomplete PACT image analysis, which was caused by the tumor boundary exceeding 5 cm in depth. The remaining 148 patients, with 162 tumors, underwent systematic scanning by HDMI, and the imaging data were batch processed (batch-processed population). Subsequently, 142 patients (age range, 23 to 73 years; mean age, 46 years) with 155 masses were validated through biopsy or postsurgical histopathology, establishing the “ground-truth” population at the mass level for developing a diagnostic model. A total of six patients with seven masses were not biopsied in the study, while clinical collaborators provided follow-up results for five of these patients (six masses) 6 months after HDMI scanning, confirming that all six masses remained classified as BI-RADS 3. These six follow-up masses were subsequently included in the ground-truth population with benign tags. To develop a diagnostic model based on the dual-modal data, we selected 77 masses from this population for supervised training of the model. The trained model was then tested on the remaining 84 masses (testing population) in 77 patients to assess its accuracy without prior knowledge of the ground truth or BI-RADS classifications (table S1 and Materials and Methods). Because the HDMI research group was not completely blinded to the BI-RADS classifications of the five follow-up patients and the four patients eligible for neoadjuvant chemotherapy, their masses were included in the training dataset.

**Fig. 3.**
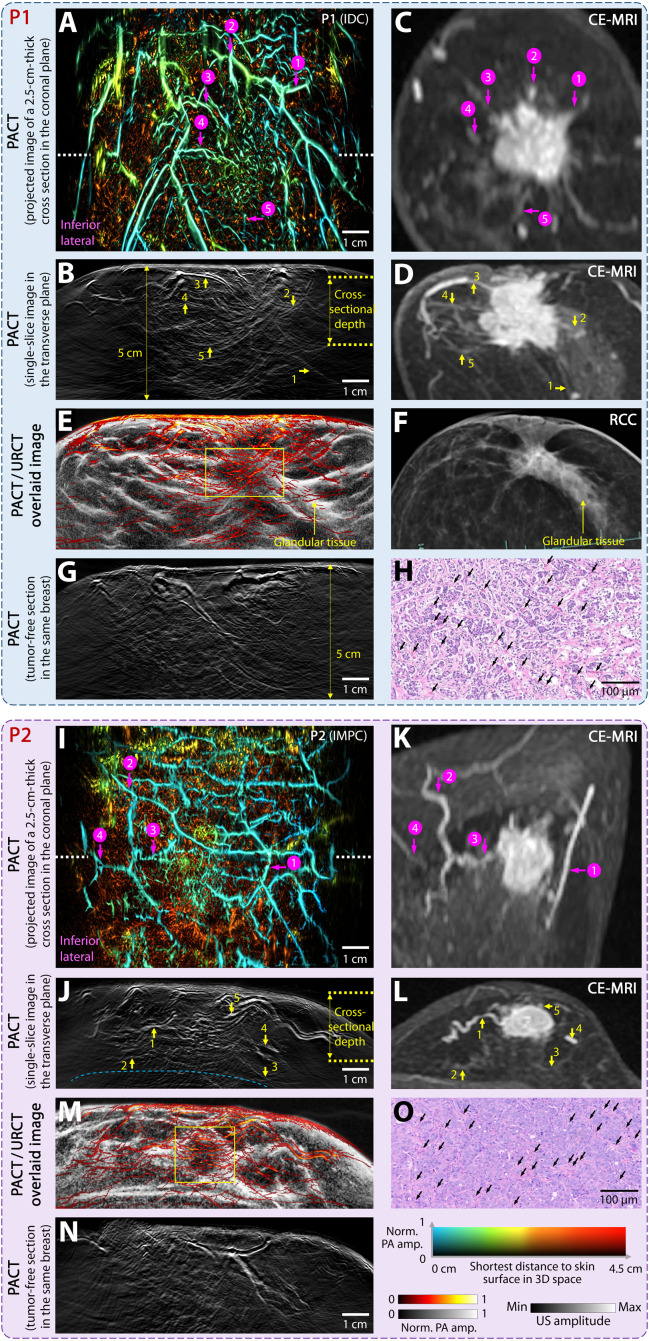
Comparison between HDMI and other modalities of the affected breasts in patients. P1 (top): invasive ductal carcinoma (IDC; grade 3); P2 (bottom): invasive micropapillary carcinoma (IMPC). (**A** and **I**) MAP images of 2.5-cm-thick cross sections [yellow dashed lines in (B) and (J)] on the coronal plane, visualizing dense and polymorphic blood vessels within tumors and surrounding peripheral radial vessels. Vasculature colors represent the shortest distance of each voxel to the skin surface in 3D. The same depth-encoded color map was applied to all MAP images to ensure consistency. (**B** and **J**) Real-time cross-sectional PACT of the breast on the transverse plane [white dashed lines in (A) and (I)]. (**C** and **K**) CE-MRI of the same breast cross sections as in (A) and (I) with dynamic postcontrast sequences. Correlated blood vessels were annotated using magenta arrows based on their similar positions and orientations. HDMI reveals more angiographic details at a faster speed and without contrast agent injection. (**D** and **L**) CE-MRI of the same breast on the transverse plane for comparison with (B) and (J). Compared to CE-MRI, where the breast was naturally sagged, the breast appears more compressed in HDMI, with angiographic features positioned closer to the chest wall. (**E** and **M**) Fused images of PACT and corresponding URCT, with PACT vascular features displayed in a hot color map overlaid on grayscale ultrasound anatomy. Tumor-affected regions are outlined with yellow boxes. (**F**) X-ray mammogram of the same breast in right craniocaudal (RCC) view as in (E), with correlated glandular structures indicated by yellow arrows (P2 did not undergo mammography). (**G** and **N**) PACT of tumor-free sections in the same breast on the transverse plane, serving as a control for the tumor-containing sections in (B) and (J). (**H** and **O**) Histologic correlations for angiogenesis within the tumors. Some microvessels are close enough together that they are volume averaged in PACT images.

**Fig. 4.**
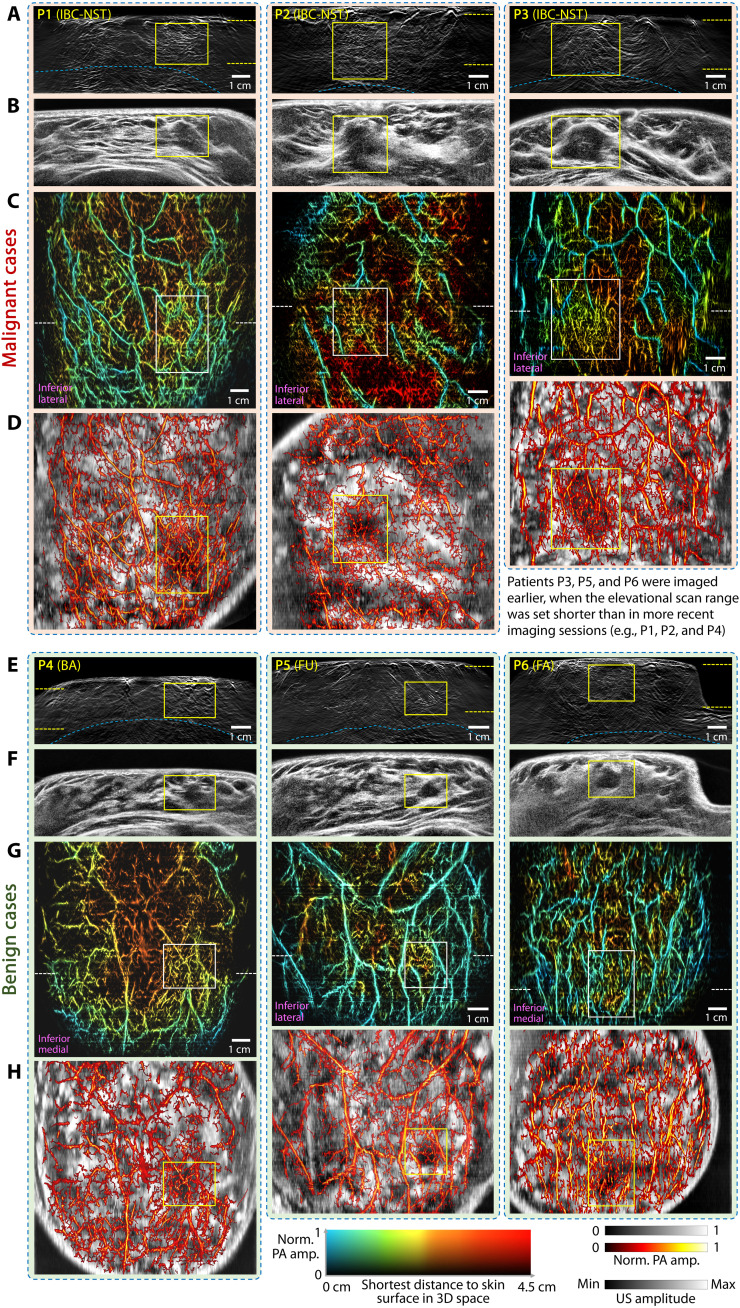
HDMI of patients with breast tumors. (**A** and **E**) PACT images of tumor-containing slices on the transverse plane, with tumor-affected regions labeled by yellow boxes and the chest walls outlined by blue dashed lines. Images are cropped either near the chest wall or at a depth of 5 cm. (**B** and **F**) URCT of the same cross sections as shown in (A) and (E), demonstrating concordance in tumor locations. (**C** and **G**) PACT images of breast cross sections [marked by yellow dashed lines in (A) and (E)] on the coronal plane, with tumors enclosed by white boxes. The white dashed lines indicate the position of the transverse cross sections shown in (A) and (E) and (B) and (F). (**D** and **H**) Fused images of PACT and URCT on the coronal plane, with PACT vascular features displayed in a hot color map overlaid on the grayscale ultrasound anatomy. The tumor-affected regions are outlined with yellow boxes. BA, breast adenosis; FA, fibroadenoma; FU, maintained BI-RADS 3 during a 6-month follow-up; IBC-NST, invasive breast carcinoma of no special type.

To ensure measurement objectivity and establish statistical significance, we scanned every patient within the “batch-processed” population by HDMI in a standardized manner, with each session lasting ~1.5 min for preparation (e.g., changing clothes) and 1.5 to 2.0 min for multiple 12-s scans. Typically, one to two scans of each breast were conducted per session. Following each scan, cross-sectional images on the transverse plane were integrated to form a volumetric image (movies S3 and S4 and Materials and Methods), which could be displayed on either the transverse or coronal plane (Figs. 3 and 4). Similar to clinical B-mode ultrasonography, the penetration depth of HDMI can be reliably quantified in the cross-sectional images on the transverse plane, which is perpendicular to the skin surface. Because no recruited patient had a tumor extending into the chest wall, we cropped the transverse images either to a depth of 5 cm or to the chest wall, as indicated by the corresponding URCT images.

To compare HDMI findings with conventional approaches, we present images from two patient with invasive ductal carcinoma (P1) and invasive micropapillary carcinoma (P2) in Fig. 3. For both patients, tumor-associated angiogenesis was clearly visible in both the coronal (Fig. 3, A and I) and transverse (Fig. 3, B and J) planes. A side-by-side comparison with CE-MRI (Fig. 3, C, D, K and L) confirmed the tumor position and surrounding major blood vessels. By overlaying the PACT angiogram on URCT anatomy (Fig. 3, E and M), we observe tumor positions and glandular tissue structures that are comparable to those seen in mammography (Fig. 3F; P2 did not undergo mammography). As a control, PACT of a tumor-free section in the same breast is also shown in (Fig. 3, G and N), displaying fewer dense, tortuous vessels than in the tumor-containing section. This proliferative blood vessel growth within the tumor-affected region was confirmed by postsurgical histopathology (Fig. 3, H and O). Compared to CE-MRI and mammography, HDMI revealed smaller vessels and tumor anatomy without the need for contrast agent injection, lengthy scan times, ionizing radiation, or painful compression.

Figure 4 illustrates cross-sectional images of six patients with breast tumors, categorized into malignant (top three, P1 to P3) and benign groups (bottom three, P4 to P6). Notably, instead of presenting the whole-breast projection, the color-encoded PACT images on the coronal plane show tumor-containing cross sections, as indicated by the yellow dashed lines in the corresponding transverse images. This accounts for the differences in color distributions across the coronal images. The depth of the cross section was selected to ensure that tumor-associated angiogenesis was not obscured by shallower or deeper blood vessels, while also displaying some blood vessel networks near the skin for visual aesthetics. To maintain consistency and facilitate cross-figure comparisons, we applied the same color map across all relevant panels, regardless of the thickness of the projected section. Accordingly, differences in color and vascular distributions in these tumor-containing sections reflect natural variations in depth and anatomy. Additional HDMI images of the batch-processed population (fig. S13) further validated the reliability of cross-sectional imaging quality on the transverse plane.

The integration of URCT and PACT in HDMI enables the simultaneous assessment of tumor-associated morphology and angiogenesis to aid in diagnosis. For example, in Fig. 4 and fig. S13, malignant lesions typically exhibit more irregular shapes and heterogeneous internal echoes in URCT images compared to benign tumors. In addition, PACT images of malignant lesions often reveal more intertwined vessels inside the tumor and more pronounced angiogenesis both within and near the boundary zones, serving as distinguishing features between malignant and benign cases. Because of challenges in accurately calculating or predicting optical fluence within deep breast tissue, quantitative measurement of blood sO_2_ is prone to inaccuracy and unreliability (*21*, *47*). Therefore, we used a single wavelength of 1064 nm to assess the distribution of relative hemoglobin concentration at similar depths near the tumor site, referred to as the “1064-amp factor” (see Materials and Methods). At this wavelength, the extinction coefficient of HbO_2_ exceeds that of deoxyhemoglobin by approximately 13-fold (fig. S7B) (*48*), and no other endogenous components in breast tissue exhibit comparable absorption to hemoglobin (*49*). Theoretically, the resulting PA signal amplitude should predominantly reflect the absorption of HbO_2_. From a physiological perspective, studies have reported elevated metabolic activity in the early stages of malignant tumors compared to benign lesions, often accompanied by increased HbO_2_ levels associated with tumor angiogenesis (*50*, *51*).

Given the capability of producing real-time breast cross-sectional images on the transverse plane using PACT and URCT, we developed a diagnostic model based on cross-sectional images that incorporate both anatomical structures and hematogenous characteristics. This model aims to facilitate real-time diagnostic suggestions or references in the future. Another reason for using transverse images for diagnosis is the reduced motion artifacts, which remain negligible even when the breast undergoes deformation during scanning. Therefore, we selected the transverse slice displaying the longest mass axis to assess hematogenous and anatomical characteristics across three specific regions defined in URCT images (fig. S13 and Materials and Methods) (*39*): the tumor zone (identified by the hypoechoic central core of the mass), the boundary zone (identified by the indistinct echogenic halo commonly found around invasive tumors), and the reference zone (positioned outside the boundary zone but at the same depth as the tumor).

**Fig. 5.**
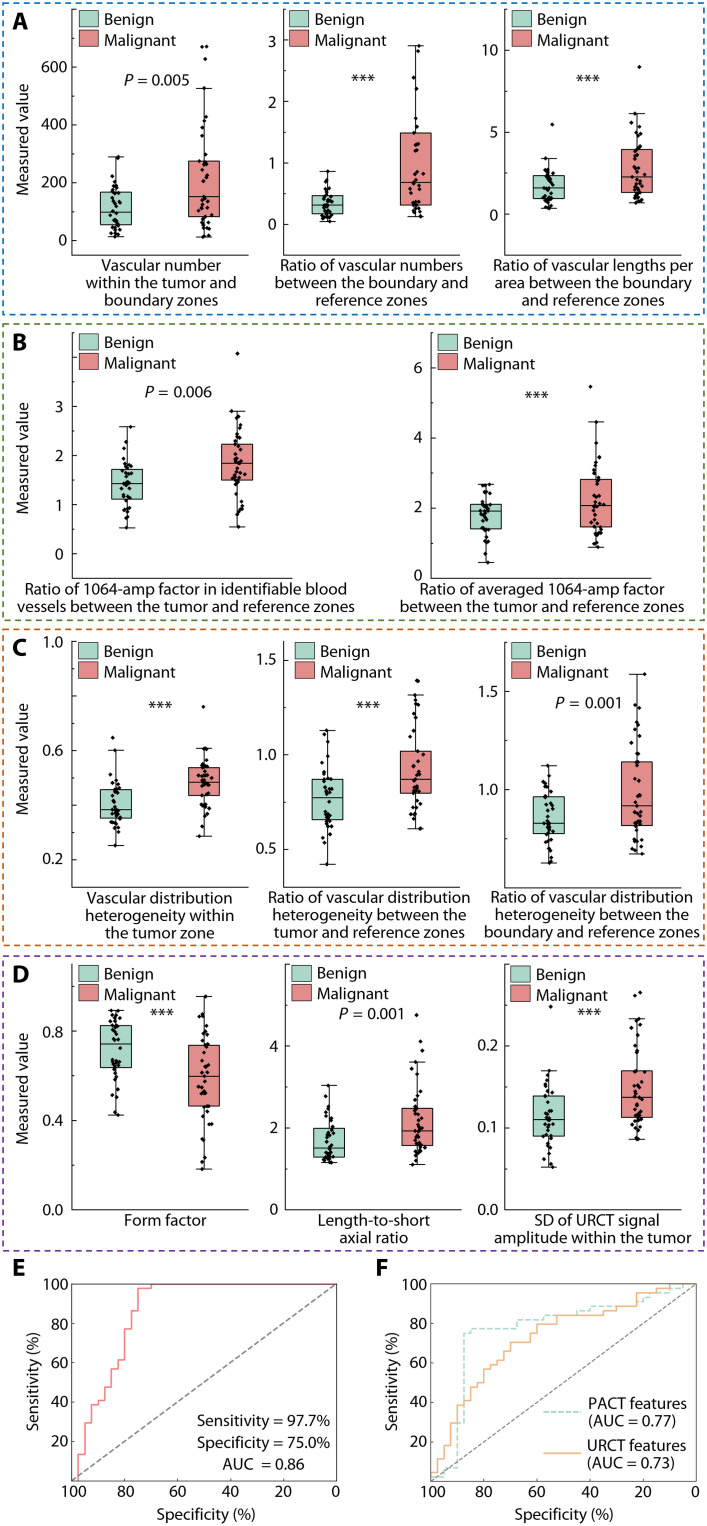
Error-bar plots differentiating benign and malignant breast masses using HDMI image features. (**A**) PACT image features related to blood vessel number and density. (**B**) PACT image features related to the PA signal amplitude (i.e., 1064-amp factor). (**C**) PACT image features related to the heterogeneity of vascular distributions. (**D**) URCT image features related to tumor anatomy. (**E**) ROC curve of HDMI by varying the POM calculated by the diagnostic model based on CNN, achieving a sensitivity of 97.7% and a specificity of 75.0%. (**F**) ROC curves of the CNN diagnostic model using either PACT features alone or URCT features alone, indicating that both imaging modalities notably contribute to breast tumor diagnosis. ***P < 0.001. *n* = 77 masses in (A) to (D); *n* = 84 masses in (E) and (F).

A total of 204 image features were quantified and evaluated in the HDMI images, revealing 11 image features (8 from PACT and 3 from URCT; Fig. 5, A to D, and table S2) with statistically significant differences (two-sided *P* < 0.01) between benign and malignant masses. We strictly controlled the number of features included in the diagnostic model for three reasons: (i) High-dimensional data are susceptible to overfitting, especially in small-sample scenarios. Eliminating irrelevant or noisy features improves the model’s ability to generalize. (ii) Through independent sample *t* tests, correlation analysis, and probability density function analysis for benign and malignant tumors (see Materials and Methods), we removed features that were irrelevant to the model, thereby enhancing its performance. (iii) Retaining only the key features simplifies the model structure, making it easier to analyze each feature’s contribution to the model’s predictive output (fig. S14).

### Statistical analysis

The primary objective of this study was to determine the early diagnostic sensitivity and specificity of HDMI compared to clinical methodologies, specifically handheld ultrasound, which is primarily used for populations with radiographically dense breasts. Following HDMI scans, the ground-truth population underwent biopsies or surgeries per clinical recommendations. Pathologists reviewed all histologic specimen slides prepared with standard sectioning and staining procedures, ultimately determining the “ground truth.” Within the ground-truth population, histopathology analysis determined 70 (43.5%) masses to be benign, 85 (52.8%) to be malignant, and 6 (3.7%) masses remained classified as BI-RADS 3 by clinical ultrasound after a 6-month follow-up. Considering that only 10.9% of recruited participants presented with multiple masses, all masses were modeled as independent observations (table S3). Using the HDMI cross-sectional images of 77 masses along with their corresponding histopathological or follow-up reports, we developed a diagnostic model based on a convolutional neural network (CNN). This model was trained in a supervised manner using 11 HDMI features for each mass (table S2 and Materials and Methods). Subsequently, the model underwent blinded testing on the remaining 84 masses (testing population) without prior knowledge of the clinical classification or the pathological ground truth.

For the purpose of early diagnosis, each mass in the testing population was classified as either benign or malignant, with a probability of malignancy (POM) assigned to each mass. We then plotted the receiver operating characteristic (ROC) curves of HDMI by varying the POM threshold from 0 to 100% (Fig. 5E). To ensure high diagnostic sensitivity above 95% (*52*), a threshold of 0.36% POM produced a diagnostic sensitivity (true-positive rate) of 97.7% [43 of 44; 99% confidence interval (CI), 84.3 to 100%] and a specificity of 75.0% (30 of 40; 99% CI, 53.9 to 90.0%). If a diagnostic sensitivity of 100% is required, then HDMI can still provide a specificity of 70.0% (28 of 40; 99% CI, 48.6 to 86.6%). In comparison, clinical ultrasound achieved a diagnostic sensitivity of 100% (44 of 44; 99% CI, 88.66 to 100%) and a specificity of 22.5% (9 of 40; 99% CI, 8.4 to 43.4%) for the same population (table S4).

Because most patients included in this study underwent biopsy or surgery, 89.3% of masses (75 of 84) in the testing population were classified as BI-RADS 4 or higher by clinics. Therefore, the diagnostic specificity is inevitably lower when the pretest probability of disease is high within the recruited population. To evaluate the contributions of PACT and URCT features alone, we trained and tested the diagnostic model using only PACT features or only URCT features (Fig. 5F). The results suggest that the inclusion of hematogenous information provided by the PACT modality significantly improves diagnostic specificity, addressing one of the critical clinical needs (*6*). The overall improvement of 52.5% in specificity (*P* < 0.001) for ambiguous breast masses would substantially reduce the rate of benign biopsies and improve workflow efficiency in breast clinics.

For benign mass diagnosis, HDMI correctly downgraded 57.5% of benign masses classified as BI-RADS 4 with clinical ultrasound to benign conclusions (23 of 40) and mistakenly upgraded 5.0% of benign masses from BI-RADS 3 by clinical ultrasound to malignant conclusions (2 of 40). The number of correct downgrades (*n* = 23) was significantly higher than the number of incorrect upgrades (*n* = 2) among benign masses evaluated with HDMI. For the diagnosis of malignant tumors, HDMI correctly upgraded 4.5% of malignant tumors from BI-RADS 4a to malignant conclusions (2 of 44) and mistakenly downgraded 2.3% of malignant tumors from BI-RADS 4c to benign conclusions (1 of 44). No significant difference in diagnostic accuracy was observed on the basis of age group, mass maximum size, or center depth (table S5).

### Angiogenesis findings in contralateral breasts of patients

During HDMI scanning, we observed an increased incidence of angiogenesis in the contralateral tumor-free breasts of patients compared to those of healthy participants (fig. S15, A and B). This phenomenon has not been reported by other imaging modalities, likely due to their low sensitivity in detecting small blood vessels. Nonetheless, HDMI demonstrated a statistically significant difference in the angiogenesis occurrence rate in tumor-free breasts between participants with and without breast tumors (fig. S15C and Materials and Methods). Because biopsies were not performed on the contralateral breasts, these angiogenesis findings have not been validated by histopathology, but the statistical analysis could serve as evidence for the universality of this phenomenon. We hypothesize that the angiogenesis observed in contralateral breasts may be linked to the stagnation of meridians, which are known to play a crucial role in maintaining physiological functions (*53*–*56*). Prolonged meridian stagnation leads to blood flow disorders and subsequent blood stasis (*57*), which have been shown to be closely associated with the development of tumors, hyperplasia, and lumps (*58*, *59*). In future studies, we will investigate the correlations between angiogenesis in tumor-free breasts and systemic breast health indicators.

## DISCUSSION

We developed HDMI from a demonstration prototype to a preproduction prototype, demonstrating its capability in early diagnosis with statistical significance. HDMI solely combines dark-field illumination, a large-aperture ultrasonic array, and relatively consistent breast shaping to provide deep breast imaging with enhanced clarity and reliability. The integration of low-noise preamplification channels and ultrasound emission circuits allows PACT and URCT to operate using the same ultrasonic array and DAQ module, generating breast images with dual contrast mechanisms simultaneously. In addition, the vertically placed half-ring ultrasonic array facilitates snapshot PACT of breast cross sections in the transverse plane without motion artifacts, achieving painless imaging of breasts ranging from cup sizes A to F and revealing tumor-associated angiogenesis and acoustic heterogeneity beyond the chest wall. The smooth scanning of the participant further extends the FOV to the entire breast, producing volumetric dual-modal images. To enhance the representation of tumor locations within the breast, we applied an image registration algorithm and presented maximum amplitude projection (MAP) images of tumor-containing cross sections in the coronal plane.

Compared to the first-generation PACT system [single-breath-hold PACT (*22*, *23*, *36*)] developed by the corresponding author before his independence, the HDMI system offers enhanced visualization of tissue near the chest wall, finer elevational resolution, deeper penetration, and the addition of the URCT modality. In comparison to the second-generation PACT system developed with his advisor (*24*), as well as representative breast PACT systems based on hemispherical detection matrices (*25*–*28*, *37*), the HDMI system takes a step closer to clinical translation, simultaneously offering real-time dual-modal imaging, clear visualization of deep tumor angiogenesis, whole-breast scanning within seconds, high reliability, reduced space requirements, and more affordable costs. Specifically, movie S2 demonstrates the detection of blood vessels up to 5 cm deep, with deeper vasculature near the chest wall exhibited greater motion than superficial vessels. This depth-dependent dynamic behavior further supports the physiological origin of the visible features, as reconstruction artifacts are unlikely to exhibit such motion correlated with both heartbeat and anatomical depth.

Rather than competing on isolated technical parameters with other PACT or ultrasound imagers, the primary goal of this study is to develop a balanced imaging platform that addresses clinical challenges, particularly the limited specificity in early breast tumor diagnosis. To ensure reliable and reproducible tumor imaging quality while systematically scanning and batch-processing data from hundreds of patients, we designed HDMI with a focus on tumor imaging clarity, performance reliability, and diagnostic accuracy. Consequently, HDMI prioritizes snapshot 2D imaging to visualize tumor-associated vasculature in detail and performs whole-breast scanning within a single 12-s breath hold, at the cost of sacrificing isotropic resolution in 3D space. We opted not to incorporate multiwavelength illumination into HDMI for three key reasons: (i) The energy limitations of affordable wavelength-tunable lasers restrict penetration depth and FOV with a single laser pulse; (ii) scanning across multiple wavelengths would decrease imaging speed, potentially introducing motion artifacts that could obscure the small and tortuous blood vessels associated with tumors; and (iii) there is currently no reliable PACT technique for accurately quantifying sO_2_ in deep biological tissues (*21*, *47*).

In summary, HDMI offers high imaging speed, a semipanoramic FOV, standardized scanning and batch data processing, ionization-free radiation, and rich imaging contrasts that simultaneously encompass anatomical and hematogenous details of the human breast without requiring exogenous contrast agent injection. Consequently, HDMI can be transformed into a powerful tool for breast tumor screening, early diagnosis, and assessment of treatment response. We begin by demonstrating HDMI’s capability for malignancy diagnosis with statistical significance. Early diagnosis, which is critical for clinics to formulate subsequent workflows, often suffers from low specificity due to the limited imaging contrast (i.e., information). Notably, the combination of imaging contrasts from PACT and URCT enabled HDMI to classify breast masses with significantly higher specificity, increasing from 22.5% (clinical ultrasonography) to 75.0% (HDMI).

In addition to the low specificity in early breast tumor diagnosis, there is currently no reliable noninvasive imaging modality widely accepted in clinical practice for closely monitoring breast cancer response to neoadjuvant chemotherapy. Because evaluating tumor responses relies on serial accurate diagnoses with measurements of anatomical and physiological features at consistent positions throughout therapy, HDMI holds the potential to meet this critical need by acquiring information similar to that of CE-MRI plus ultrasonography, yet with high imaging speed, standardized scanning, and endogenous contrast. In addition, studies have demonstrated that PACT of the breast can noninvasively distinguish molecular subtypes (*40*), paving the way for broader applications of HDMI. Leveraging PACT’s high sensitivity to small blood vessels, integrating PACT with ultrasonography may also enhance detection sensitivity in early breast cancer screening. Instead of competing with MRI, mammography, or ultrasonography, we propose HDMI as a prototype complementary to existing modalities, addressing clinical needs that current methods do not fully meet.

## MATERIALS AND METHODS

### System construction

The primary intention behind designing HDMI was to simultaneously provide high-quality PA and ultrasound images of breast cross sections in real time with high reliability. While developing the system based on a hemispherical array or detection matrix could ensure more uniform resolution in 3D space (*24*, *37*), it would compromise the FOV of snapshot PACT with affordable channel numbers (*60*). In addition, clinics are accustomed to reviewing cross-sectional images containing lesions, similar to the approach used in x-ray computed tomography and MRI, rather than directly analyzing a 3D-rendered image of the whole breast. Consequently, HDMI uses a cylindrically focused half-ring array to deliver dual-modal images of arbitrary breast cross sections in the transverse plane at a 10-Hz frame rate and allows for high-speed standardized scanning of the entire breast within 12 s. According to the American National Standards Institute (ANSI) safety limit (*61*), a 10-Hz laser repetition rate offers optimal signal-to-noise ratio for a given average laser power. In the array with a 3-MHz center frequency (customized by Imasonic Inc.), 512 transducer elements are nonuniformly distributed along a 176° arc with a 130-mm radius (fig. S16), creating an elliptical FOV with an 82-mm-long axis and a 63-mm-short axis according to the spatial Nyquist criterion (*60*). The mechanical focalization of each element results in an acoustic focus of ~2.3 mm with a field depth of 65 mm. The slit in the middle of the breast-shaping mold is substantially wider than the acoustic diffraction–limited field.

Another advantage of using the half-ring array is the flexibility it provides for designing the light path for illumination. On the basis of our experience with developing earlier generation PACT systems (*23*, *24*), we observed artifacts in deep tissues caused by strong surface PA signals, which can be two orders of magnitude higher than those from tissues beyond 2 cm in depth (*62*). To address this issue, we adopted the dark-field illumination method from acoustic-resolution PA mesoscopy (*44*). This method involves depositing ~80 to 90% of the light energy on skin surfaces outside the acoustically defined imaging plane (Fig. 1D). Because of space constraints for patient imaging, we modified the demonstration prototype (fig. S1B) by replacing the single large laser (Nimma 2000, Beamtech Optronics Co. Ltd.) with two smaller lasers (Nimma 900, Beamtech Optronics Co. Ltd.) to provide comparable optical fluence and enclosed their power supplies within the preproduction prototype (Fig. 1 and fig. S1C). Instead of combining the two laser beams, we aligned them in parallel to two engineered diffusers (EDR-20×10, RPC Photonics Inc.) to expand the laser beams into two rectangular illumination areas with a total length of ~10 cm and a width of 2.2 cm on the skin surface (fig. S1A), resulting in an optical fluence of ~60 mJ/cm^2^, which is safely below the ANSI limit (*61*). Because the projected light pattern on the skin surface is much broader than the acoustically defined imaging cross section (~2.3 mm), moderate variations in skin conformity are unlikely to produce substantial changes in the optical distribution within the imaging plane. Although we initially designed two optical windows for dual-side illumination (Fig. 1D), we opted to use only one window after considering the following factors: (i) The dual-side laser beams may partially overlap when imaging smaller breasts (with the skin surface positioned further from the window), potentially causing localized optical fluence to exceed the ANSI safety limit; and (ii) the inner-side optical paths may interfere with tens of electrical cables.

In addition to reducing artifacts induced by strong surface PA signals, the implementation of dark-field illumination also alleviates the demand on the dynamic range of DAQ systems. In a 12-bit DAQ system, for example, electrical background noise typically occupies 2 to 4 bits (depending on gain settings), providing an effective amplitude ratio range of 2^8^ = 256 between the strongest signal from the skin surface and the weakest signal from deep tissue. Consequently, if the PA signal amplitude varies excessively within the FOV, then the DAQ may either saturate the strong signals or bury weak signals in background noise (without considering signal averaging). While bright-field illumination offers better overall efficiency (fig. S3), its optical fluence variation along depth is substantially larger than that of dark-field illumination (220-fold versus 62-fold). In addition, larger blood vessels near the skin surface may further exacerbate the disparity of PA signal amplitudes to ~1000-fold. If signals are cropped because of DAQ saturation or compromised by electrical noise, reconstruction algorithms cannot effectively recover the information they carry.

As illustrated in fig. S17, cross-sectional PACT and URCT are alternated during a comprehensive breast scan. Following each laser pulse (<10 ns), 480 ultrasonic elements and DAQ channels capture the PA signals within 160 μs. Given the pulsed lasers operate at a 10-Hz repetition rate, there is a temporal gap exceeding 99.8 ms between each PACT snapshot. During this interval, electrical pulses are sequentially transmitted from a custom-developed ultrasound transmission board to 30 evenly distributed elements within the ultrasonic array. To safeguard the array when the coupling water surface is below the top elements, the top two elements (the first and last in the array) are excluded from receiving electrical pulses. For URCT of a breast cross section, each transmission element is sequentially excited, followed by simultaneous signal reception by the remaining 480 transducer elements. Consequently, each URCT cross-sectional image is generated by compiling 30 frames, each excited from a different angle. The frame number used to compose each URCT image was limited by the data streaming bandwidth of the DAQ system. The entire process takes 93 ms, during which the breast moves a distance (≤0.93 mm) less than half the beam width of the acoustic focus. While multiangle plane wave compounding could enhance the signal-to-noise ratio of the ultrasound image, its implementation would require electrical switches between every transducer element and the preamplifier channels (necessary for high-quality PACT). Since PA signals from the deep tissue are typically much weaker than ultrasonography signals, we chose to exclude electrical switches in the prototype to avoid introducing potential noise and interference into the PA signals. Consequently, in the HDMI system, the transducer elements were exclusively dedicated to either transmission or reception, without switching between these modes.

In addition to the key components of optical illumination, ultrasound detection, and electrical signal transceiving in the HDMI system, multiple supplementary modules and designs are essential to ensure smooth and reliable operation. For instance, we used the breast-shaping mold to standardize the positions and shapes of the breasts. To accommodate larger breasts (≥C cup) within the mold, one or two soft pads were placed near the imaging aperture under the bed sheet to slightly elevate the chest wall. To maintain the coupling water level for different breast sizes and keep the water temperature consistent at 36°C throughout a full day of imaging, we installed two water tanks equipped with a heater and pump for water circulation. Another critical component of the HDMI system is the mechanical framing and motion system, which supports all modules and ensures smooth scanning of the imaging platform. The entire HDMI system is supported by six Footmaster casters, except for the laser power supplies, which are supported by their own wheels. In addition, since all modules are housed within the HDMI enclosure, we incorporated heat dissipation fans and noise insulation foam to effectively manage thermal and acoustic conditions.

### Imaging protocol and clinical testing procedures

All human imaging experiments adhered to relevant guidelines and regulations. The protocol was approved by the Ethics Committee of Zhejiang University (2023-001) and the Clinical Research Ethics Committee of the First Affiliated Hospital, Zhejiang University School of Medicine (IIT20230241B). Participants provided verbal consent to participate and signed informed consent forms, which were securely stored. All HDMI data files were anonymized using participant acronyms and imaging dates and were stored separately from medical history files to maintain confidentiality. The authors in this study were divided into two groups: the HDMI research group and the clinical group. The clinical group was responsible for acquiring patient information, obtaining signed consent forms, and referring patients to the HDMI group. They provided tumor location information (limited to the breast side only) without offering any diagnostic comments. Clinical diagnostic information, including the BI-RADS score, was shared with the HDMI group only after they decided to temporarily cease patient imaging for model training or testing. As a result, the HDMI group, clinical BI-RADS scorers, and pathologists were triple-blinded to each other during HDMI scanning and data processing. Notably, HDMI identified a benign mass in the right breast of a healthy volunteer (Fig. 4, P5), who was unaware of its presence before the HDMI scan. The HDMI research group suggested a follow-up in the clinic, where the mass was later confirmed as a BI-RADS 3 nodule. This classification remained unchanged after 6 months. For clinical ultrasound, BI-RADS 4a or higher are classified as malignant cases since a biopsy is typically required.

Clinical testing of the preproduction prototype was conducted in an imaging room at Zhejiang University School of Medicine, from 10 July 2023 to 2 December 2024. Study with the preproduction prototype included 16 healthy female volunteers and 158 female patients (170 total, with 12 excluded) with breast masses. Healthy volunteers were adult women with no palpable breast masses and no lesions classified as BI-RADS 2 or higher within 3 months before participation. Eligible patients were women over 18 with breast masses assessed as BI-RADS 3 to 6, recommended for follow-up, biopsy, or surgery. Participants with more than three suspicious masses were excluded to ensure the independence of each mass. Additional exclusions included posttraumatic ecchymosis, biopsies within 7 days, or surgeries in the same breast quadrant within the past 18 months (*39*). Enrolled participants signed consent forms, were briefed on imaging procedures and safety precautions, and changed into disposable test clothing before imaging. PTFE/PVC film and bed sheets were replaced for each participant. Each breast was scanned one to two times with slight adjustments to positioning after each scan.

During the preparation of volumetric images, we noticed that some large breasts experienced slight deformations due to friction with the breast-shaping mold during scanning, which compromised the clarity of images in the coronal plane. While image registration between each snapshot transverse image helped mitigate this issue, it still affected the reliability of generating high-quality volumetric images. To address this, we replaced the flexible PTFE film with harder PVC films starting in August 2024. This change minimized breast deformation by reducing the shear force from the friction, resulting in more reliable volumetric image quality. Initially, the elevational scan range was set to 85 mm for the first approximately two-thirds of patients but was later increased to 120 mm with a faster scan speed to accommodate off-centered breast positioning. The scanning step was adjusted from 0.71 to 1 mm, which remains less than half of the elevational resolution (fig. S10B).

### Real-time dual-modal image reconstruction

During patient scanning, cross-sectional PACT and URCT images were reconstructed in real time and simultaneously displayed on the transverse plane (movie S1). Given the laser’s 10-Hz repetition rate, “real time” in this manuscript refers to the ability to display a cross-sectional image within 0.1 s. We used a three-thread algorithm for parallel data processing: (i) Sampling thread acquired PACT and URCT data from DAQs and loaded it into a circular queue. The use of apodization windows helps mitigate artifacts associated with the semipanoramic detection angle coverage. (ii) Real-time PACT reconstruction thread retrieved PACT data and executed the PACT image reconstruction algorithm. (iii) Real-time URCT reconstruction thread retrieved URCT data and executed the URCT image reconstruction algorithm. To reveal optical absorbance in PACT images, the reconstruction algorithm performed a direct weighted summation of time-shifted PA signals from all reception elements in the array. Given that warm water for acoustic coupling has a speed of sound around 1.52 mm/μs, which lies between those of glandular and adipose tissues (*63*), a single-speed DAS algorithm was used to ensure reliable performance (*45*).

URCT image reconstruction used the synthetic aperture ultrasound algorithm (*64*). To avoid artifacts from transmitted ultrasound signals, only reflection signals from elements with an axis direction less than 90° relative to the transmitted element’s axis were used. After applying the Hilbert transformation to the original signal, a DAS algorithm (*65*) with incoherent compounding was performed to improve the signal-to-noise ratio of the reconstructed image. The reconstructed cross-sectional image on the transverse plane had dimensions of 12 cm by 5 cm, with the image midpoint ~5 mm beneath the array’s circular center. Each transverse image was reconstructed into 480 pixels by 200 pixels to ensure smooth reconstruction and display during scanning. We used a Xeon series server with an Intel(R) Xeon(R) W7-2475X 2.59-GHz central processing unit and an NVIDIA GeForce RTX 3090 graphics processing unit (GPU) for real-time dual-modal image reconstruction. The threading configuration was optimized for GPU Compute Unified Device Architecture (CUDA) by assigning each block within the CUDA grid 40 by 20 threads, resulting in a grid structure of 12 blocks along the *x* axis and 10 blocks along the *y* axis, enhancing parallel computing capacity for reconstruction.

While we used DAS reconstruction in this study due to its time efficiency and reliability, more advanced reconstruction approaches could further enhance image quality. For instance, the model-based iterative reconstruction algorithm (*37*, *66*, *67*) formulates an optimization problem to iteratively minimize the error between the reconstructed image and the initial PA pressure, directly mitigating artifacts caused by strong surface signals. In general, iterative reconstruction algorithms are computationally demanding and require considerable time to reconstruct each image. Alternatively, deep learning–based PACT reconstruction algorithms have been developed to address issues such as the limited bandwidth at tissue boundaries (*68*), correct artifacts from acoustic reflections at these boundaries (*69*), and improve images obtained from sparse sampling or limited-view angles (*70*).

### Image batch reconstruction and processing

Given the interpretative challenges posed by the bipolar images produced by the conventional DAS approach (fig. S6), we displayed the images in this manuscript using a unipolar weighted DAS method (*45*, *46*), which combines the original bipolar image with its derivatives to compensate for the negative slope of the bipolar signal. Because negative signals in unipolar images are highly suppressed, the visibility of vascular structures was enhanced on the cleaner background (figs. S5 and S6). For offline reconstruction, pixel size was refined to 0.1 mm by 0.1 mm. Spatial interpolation was applied to mitigate aliasing artifacts caused by spatial undersampling outside the well-resolved FOV. After reconstructing the cross-sectional images on the transverse plane, we applied an image postprocessing algorithm to each image. For PACT images, we first applied adaptive depth compensation to enhance weak signals from deep and fine vasculature. Subsequently, Hessian-based Frangi vesselness filtration (*71*) was implemented to enhance the contrast of blood vessels with diameters ranging from 2 to 7 pixels. The filtered slices were weighted with a factor of 0.1 and superimposed onto the normalized original image. Side-by-side comparisons demonstrated that depth compensation and vesselness filtration did not introduce additional features to the original image (fig. S18). The contrast-to-noise ratio (CNR) calculation in Fig. 2E was performed using the unfiltered original images. No amplitude thresholding was applied to PACT images, except for those overlaid on their URCT counterparts. For URCT images, logarithmic compression, dynamic range selection, and contrast-limited adaptive histogram equalization (*72*) were applied to help improve the CNR and refine image quality.

Volumetric images of the breast were generated by stacking cross-sectional images taken on the transverse plane. The distance between cross sections varied from 0.71 to 1.0 mm, depending on the scanning speed. To minimize motion-induced artifacts during scanning, image registration was applied to each cross-sectional image. Interpolation was then performed along the scanning direction to ensure uniform voxel dimensions in the volumetric images. To further improve image sharpness along the scanning direction (fig. S4), we used a super-resolution method based on a deep convolutional network with 20 weighted layers (*73*). This network estimated the value of each central pixel by leveraging contextual information from a large receptive field. Afterward, Hessian-based Frangi vesselness filtration was applied for further refinement (fig. S11). The pseudocode for image reconstruction and postprocessing is provided at the end of the Supplementary Materials.

### Quantitative analysis of HDMI images

Before conducting the quantitative analysis, several data preprocessing steps were undertaken: (i) Image origin adjustment: The reconstruction origin was adjusted to position the bottom skin surface 3 to 5 mm above the image bottom. (ii) Zone segmentation: Specific zones in the URCT images were delineated (fig. S13) (*39*) and subsequently transferred to the corresponding locations on the coregistered PACT images. The tumor zone was identified by the hypoechoic region of the mass, the boundary zone was defined as the indistinct echogenic halo surrounding the mass, and the reference zone was delineated equidistant from the skin surface as the tumor, positioned more than 5 mm away from the outer edge of the boundary zone. (iii) Blood vessel skeleton extraction: Gaussian filtering and Hessian-based Frangi vesselness filtration (*71*) were used to enhance and segment blood vessels. Vessel skeletons were further extracted using morphological operations (*74*), including the removal of small objects, filling of small holes, elimination of branch points, and clearing of small spurs.

To develop a diagnostic model based on HDMI images, we performed a quantitative analysis of PACT and URCT images by extracting specific features such as blood vessel density, 1064-amp factor, vascular distribution heterogeneity, tumor morphology, and echo signal characteristics. In the PACT images, we quantified the number and density of blood vessels using the vessel skeleton map, assessed the distribution of 1064-nm induced PA signal amplitude in the unipolar PACT images, and evaluated vascular distribution heterogeneity by computing the SD of the vascular signal. In the URCT images, we analyzed the tumor shape and ultrasound signal characteristics. Quantitative analysis of HDMI images was conducted across the tumor zone, boundary zone, and reference zone. A comprehensive set of features was extracted from the dataset and subjected to independent sample *t* tests, correlation analysis, and probability density function analysis for model’s input feature selection:

1) Independent sample *t* tests: Each individual feature was assessed for its ability to distinguish between benign and malignant lesions. Features with two-tailed *P* values less than 0.01 were retained for the correlation analysis.

2) Correlation analysis: Pairs of features with a correlation exceeding 0.85 were considered to have overlapping predictive contributions (*75*). To ensure each feature independently contributed to the model, we only selected the features with smaller *P* values.

3) Probability density function analysis: We analyzed the overlap area between benign and malignant cases. Features with an overlap area less than 75% were retained to ensure higher discriminative power and reduce redundant information, thereby simplifying model training.

Ultimately, a subset of 11 features (Fig. 5 and table S2) that met these criteria were retained and were used as input for our CNN diagnostic model. Specifically, we explored the relevance of HDMI features (Fig. 5) to the clinical BI-RADS lexicon (*76*). The form factor (4π × area/perimeter^2^) (*77*) quantifies the similarity of the mass to a circle; this feature, along with the length-to-short axial ratio (*78*), can be correlated with the “shape of mass” descriptor in BI-RADS (*79*). The SD of URCT signal amplitude within the tumor can be associated with the “echo pattern” in the BI-RADS descriptor list. Because blood vessels typically induce ultrasound echo signals (*80*), the number and density of blood vessels in the boundary zones (PACT image feature) can be correlated, although indirectly, with the “margin of mass” and “lesion boundary” descriptors. BI-RADS also incorporates posterior acoustic features to assess tumor malignancy. However, this factor is not applicable to URCT images, as the large FOV of URCT better reveals posterior acoustic structures.

### Development of the diagnostic model

To prevent potential bias due to differing data sizes, we applied zero mean and unit variance scaling to both the training and test datasets. The diagnostic model was developed by adopting a CNN for binary classification, combining classical convolution operations with modern deep learning regularization techniques to enhance generalization and reduce overfitting. The CNN architecture consists of four 1D convolutional (Conv1D) layers, followed by two MaxPooling layers. Rectified linear unit (ReLU) activation functions are applied after each Conv1D layer. Three fully connected layers, each with ReLU activations and a dropout rate of 0.5, help reduce the risk of overfitting. The output layer uses a Sigmoid activation function to produce a probability estimate (i.e., POM) for the binary classification task.

We further assessed feature importance using permutation importance (*81*), which quantifies the contribution of each feature to the model’s performance. This is done by measuring the change in performance when the values of a specific feature are randomly permuted. The performance decrease is indicated by the change in the area under the ROC curve (AUC) before and after the random permutation. Through this method, we generated 11 ROC curves and calculated their AUCs (fig. S14). The SD of URCT signal amplitude within the tumor and vascular number within the tumor/boundary zones were found to be the two most important features for the CNN model.

### Analysis of tumor-free breasts of patients and healthy volunteers

To compare the contralateral breasts of patients with the breasts of healthy volunteers, we selected 40 contralateral breasts from patient PACT images and 32 healthy breasts from volunteer data. To minimize the confounding effects of age, we selected participants aged 24 to 45 years in both cohorts. In addition, to account for any residual age-related differences due to nonidentical age distributions, we performed an analysis of covariance (ANCOVA) with age as a covariate. For each breast, we uniformly selected 12 frames on the transverse plane at intervals of 8 to 10 mm for comprehensive analysis. To mitigate the influence of large superficial blood vessels, we excluded image features within 6 mm of the skin surface. We quantified vascular characteristics within the breast tissue and identified features that exhibited statistically significant differences (two-sided *P* < 0.01) between the tumor-free breasts of patients and healthy volunteers. The identified features include total blood vessel number, angiogenesis occurrence rate, and vascular distance metric (*74*). Specifically, angiogenesis occurrence was determined by counting nonoverlapping 4 mm–by–4 mm regions with vascular density exceeding 1.5 times the mean value of the entire breast. The vascular distance metric was defined as the ratio of the straight-line distance between the two end points of a blood vessel to the actual path length of the vessel. The quantitative analysis of the PACT angiogram was performed using a blood vessel skeleton map, which was generated by shrinking the binarized vessel features into a skeleton and removing isolated, tiny features smaller than 5 pixels.

## Supplementary Materials

### The PDF file includes:

Figs. S1 to S18 Tables S1 to S5 Legends for movies S1 to S4 Pseudocode References

## References

[1] H. Sung, J. Ferlay, R. L. Siegel, M. Laversanne, I. Soerjomataram, A. Jemal, F. Bray, Global cancer statistics 2020: Globocan estimates of incidence and mortality worldwide for 36 cancers in 185 countries. CA Cancer J. Clin. 71, 209–249 (2021). 33538338 10.3322/caac.21660

[2] B. J. Fueger, P. Clauser, P. Kapetas, N. Pötsch, T. H. Helbich, P. A. T. Baltzer, Can supplementary contrast-enhanced MRI of the breast avoid needle biopsies in suspicious microcalcifications seen on mammography? A systematic review and meta-analysis. Breast 56, 53–60 (2021). 33618160 10.1016/j.breast.2021.02.002 PMC7907894

[3] H. R. Ferreira Dalla Pria, M. E. Scoggins, T. W. Moseley, V. Vishwa, S. Jean, S. Vuong, V. Diaz, A. Elhatw, Current status of imaging for breast cancer staging. Curr. Breast Cancer Rep. 16, 126–133 (2024).

[4] Y. Wang, Y. Li, Y. Song, C. Chen, Z. Wang, L. Li, M. Liu, G. Liu, Y. Xu, Y. Zhou, Q. Sun, S. Shen, Comparison of ultrasound and mammography for early diagnosis of breast cancer among chinese women with suspected breast lesions: A prospective trial. Thorac. Cancer. 13, 3145–3151 (2022). 36177910 10.1111/1759-7714.14666 PMC9663682

[5] N. Aristokli, I. Polycarpou, S. C. Themistocleous, D. Sophocleous, I. Mamais, Comparison of the diagnostic performance of magnetic resonance imaging (MRI), ultrasound and mammography for detection of breast cancer based on tumor type, breast density and patient’s history: A review. Radiography 28, 848–856 (2022). 35148941 10.1016/j.radi.2022.01.006

[6] M. Heijblom, J. M. Klaase, F. M. van den Engh, T. G. van Leeuwen, W. Steenbergen, S. Manohar, Imaging tumor vascularization for detection and diagnosis of breast cancer. Technol. Cancer Res. Treat. 10, 607–623 (2011). 22066601 10.7785/tcrt.2012.500227

[7] N. Sharma, M. McMahon, I. Haigh, Y. Chen, B. J. G. Dall, The potential impact of digital breast tomosynthesis on the benign biopsy rate in women recalled within the UK breast screening programme. Radiology 291, 310–317 (2019). 30888932 10.1148/radiol.2019180809

[8] R. G. Blanks, R. Given-Wilson, R. Alison, J. Jenkins, M. G. Wallis, An analysis of 11.3 million screening tests examining the association between needle biopsy rates and cancer detection rates in the English NHS breast cancer screening programme. Clin. Radiol. 74, 384–389 (2019). 30799096 10.1016/j.crad.2019.01.015

[9] L. V. Wang, S. Hu, Photoacoustic tomography: In vivo imaging from organelles to organs. Science 335, 1458–1462 (2012). 22442475 10.1126/science.1216210 PMC3322413

[10] Y. Zhou, J. Yao, L. V. Wang, Tutorial on photoacoustic tomography. J. Biomed. Opt. 21, 061007 (2016). 27086868 10.1117/1.JBO.21.6.061007 PMC4834026

[11] D. Hanahan, R. A. Weinberg, Hallmarks of cancer: The next generation. Cell 144, 646–674 (2011). 21376230 10.1016/j.cell.2011.02.013

[12] J. Folkman, Angiogenesis in cancer, vascular, rheumatoid and other disease. Nat. Med. 1, 27–30 (1995). 7584949 10.1038/nm0195-27

[13] J. E. Bluff, S. R. Menakuru, S. S. Cross, S. E. Higham, S. P. Balasubramanian, N. J. Brown, M. W. Reed, C. A. Staton, Angiogenesis is associated with the onset of hyperplasia in human ductal breast disease. Br. J. Cancer 101, 666–672 (2009). 19623180 10.1038/sj.bjc.6605196 PMC2736809

[14] P. M. Carpenter, W. P. Chen, A. Mendez, C. E. McLaren, M. Y. Su, Angiogenesis in the progression of breast ductal proliferations. Int. J. Surg. Pathol. 19, 335–341 (2011). 19403546 10.1177/1066896909333511 PMC3771508

[15] S. C. Heffelfinger, R. Yassin, M. A. Miller, E. Lower, Vascularity of proliferative breast disease and carcinoma in situ correlates with histological features. Clin. Cancer Res. 2, 1873–1878 (1996). 9816143

[16] N. B. Teo, B. S. Shoker, C. Jarvis, L. Martin, J. P. Sloane, C. Holcombe, Vascular density and phenotype around ductal carcinoma in situ (DCIS) of the breast. Br. J. Cancer 86, 905–911 (2002). 11953822 10.1038/sj.bjc.6600053 PMC2364162

[17] N. Weidner, J. P. Semple, W. R. Welch, J. Folkman, Tumor angiogenesis and metastasis - Correlation in invasive breast carcinoma. N. Engl. J. Med. 324, 1–8 (1991). 10.1056/NEJM199101033240101 1701519

[18] J. Folkman, Role of angiogenesis in tumor growth and metastasis. Semin. Oncol. 29, 15–18 (2002). 10.1053/sonc.2002.37263 12516034

[19] J. A. Nagy, S. H. Chang, A. M. Dvorak, H. F. Dvorak, Why are tumour blood vessels abnormal and why is it important to know? Br. J. Cancer 100, 865–869 (2009). 19240721 10.1038/sj.bjc.6604929 PMC2661770

[20] M. S. Gordon, D. S. Mendelson, G. Kato, Tumor angiogenesis and novel antiangiogenic strategies. Int. J. Cancer 126, 1777–1787 (2010). 19904748 10.1002/ijc.25026

[21] L. Lin, L. V. Wang, The emerging role of photoacoustic imaging in clinical oncology. Nat. Rev. Clin. Oncol. 19, 365–384 (2022). 35322236 10.1038/s41571-022-00615-3

[22] L. Lin, X. Tong, P. Hu, M. Invernizzi, L. Lai, L. V. Wang, Photoacoustic computed tomography of breast cancer in response to neoadjuvant chemotherapy. Adv. Sci. 8, 2003396 (2021). 10.1002/advs.202003396 PMC8025032 33854889

[23] L. Lin, P. Hu, J. Shi, C. M. Appleton, K. Maslov, L. Li, R. Zhang, L. V. Wang, Single-breath-hold photoacoustic computed tomography of the breast. Nat. Commun. 9, 2352 (2018). 29907740 10.1038/s41467-018-04576-z PMC6003984

[24] L. Lin, P. Hu, X. Tong, S. Na, R. Cao, X. Yuan, D. C. Garrett, J. Shi, K. Maslov, L. V. Wang, High-speed three-dimensional photoacoustic computed tomography for preclinical research and clinical translation. Nat. Commun. 12, 882 (2021). 33563996 10.1038/s41467-021-21232-1 PMC7873071

[25] Y. Matsumoto, Y. Asao, H. Sekiguchi, A. Yoshikawa, T. Ishii, K. I. Nagae, S. Kobayashi, I. Tsuge, S. Saito, M. Takada, Y. Ishida, M. Kataoka, T. Sakurai, T. Yagi, K. Kabashima, S. Suzuki, K. Togashi, T. Shiina, M. Toi, Visualising peripheral arterioles and venules through high-resolution and large-area photoacoustic imaging. Sci. Rep. 8, 14930 (2018). 30297721 10.1038/s41598-018-33255-8 PMC6175891

[26] I. Yamaga, N. Kawaguchi-Sakita, Y. Asao, Y. Matsumoto, A. Yoshikawa, T. Fukui, M. Takada, M. Kataoka, M. Kawashima, E. Fakhrejahani, S. Kanao, Y. Nakayama, M. Tokiwa, M. Torii, T. Yagi, T. Sakurai, H. Haga, K. Togashi, T. Shiina, M. Toi, Vascular branching point counts using photoacoustic imaging in the superficial layer of the breast: A potential biomarker for breast cancer. Photoacoustics 11, 6–13 (2018). 30003041 10.1016/j.pacs.2018.06.002 PMC6039965

[27] A. Oraevsky, R. Su, H. Nguyen, J. Moore, Y. Lou, S. Bhadra, L. Forte, M. Anastasio, W. Yang, “Full-view 3D imaging system for functional and anatomical screening of the breast,” in *Proceedings Volume 10494, Photons Plus Ultrasound: Imaging and Sensing 2018*, San Francisco, CA, USA, 11 April 2018 (SPIE, 2018); 10.1117/12.2318802.

[28] S. M. Schoustra, B. De Santi, T. J. P. M. Op ‘t Root, C. A. H. Klazen, M. van der Schaaf, J. Veltman, W. Steenbergen, S. Manohar, Imaging breast malignancies with the twente photoacoustic mammoscope 2. PLOS ONE 18, e0281434 (2023). 36862628 10.1371/journal.pone.0281434 PMC9980787

[29] G. Zhang, W. Li, M. Yang, C. Li, Developing a photoacoustic whole-breast imaging system based on the synthetic matrix array. Front. Phys. 8, 600589 (2020).

[30] N. Nyayapathi, R. Lim, H. Zhang, W. Zheng, Y. Wang, M. Tiao, K. W. Oh, X. C. Fan, E. Bonaccio, K. Takabe, J. Xia, Dual scan mammoscope (DSM)-A new portable photoacoustic breast imaging system with scanning in craniocaudal plane. IEEE Trans. Biomed. Eng. 67, 1321–1327 (2020). 31425013 10.1109/TBME.2019.2936088

[31] E. Zheng, H. Zhang, S. Goswami, I. E. Kabir, M. M. Doyley, J. Xia, Second-generation dual scan mammoscope with photoacoustic, ultrasound, and elastographic imaging capabilities. Front. Oncol. 11, 779071 (2021). 34869029 10.3389/fonc.2021.779071 PMC8640448

[32] T. Han, M. Yang, F. Yang, L. Zhao, Y. Jiang, C. Li, A three-dimensional modeling method for quantitative photoacoustic breast imaging with handheld probe. Photoacoustics 21, 100222 (2020). 33318929 10.1016/j.pacs.2020.100222 PMC7726342

[33] H. Zhang, E. Zheng, W. Zheng, C. Huang, Y. Xi, Y. Cheng, S. Yu, S. Chakraborty, E. Bonaccio, K. Takabe, X. C. Fan, W. Xu, J. Xia, OneTouch automated photoacoustic and ultrasound imaging of breast in standing pose. *IEEE Trans. Med. Imaging* (2025). 10.1109/TMI.2025.3578929 PMC12707420 40504720

[34] M. Heijblom, D. Piras, F. M. van den Engh, M. van der Schaaf, J. M. Klaase, W. Steenbergen, S. Manohar, The state of the art in breast imaging using the twente photoacoustic mammoscope: Results from 31 measurements on malignancies. Eur. Radiol. 26, 3874–3887 (2016). 26945762 10.1007/s00330-016-4240-7 PMC5052314

[35] Y. Asao, Y. Hashizume, T. Suita, K. I. Nagae, K. Fukutani, Y. Sudo, T. Matsushita, S. Kobayashi, M. Tokiwa, I. Yamaga, E. Fakhrejahani, M. Torii, M. Kawashima, M. Takada, S. Kanao, M. Kataoka, T. Shiina, M. Toi, Photoacoustic mammography capable of simultaneously acquiring photoacoustic and ultrasound images. J. Biomed. Opt. 21, 116009 (2016). 27893089 10.1117/1.JBO.21.11.116009

[36] X. Tong, C. Z. Liu, Y. Lou, L. Lin, J. Dzubnar, M. Invernizzi, S. D. Santos, Y. Zhang, R. Cao, P. Hu, J. Zheng, J. Torres, A. Kasabyan, L. L. Lai, L. D. Yee, L. V. Wang, Panoramic photoacoustic computed tomography with learning-based classification enhances breast lesion characterization. Nat. Biomed. Eng., 10.1038/s41551-025-01435-3 (2025). 10.1038/s41551-025-01435-3 PMC13067902 40555759

[37] M. Dantuma, F. Lucka, S. C. Kruitwagen, A. Javaherian, L. Alink, R. P. Pompe van Meerdervoort, M. Nanninga, T. J. P. M. Po ‘t Root, B. De Santi, J. Budisky, G. Bordovsky, E. Coffy, M. Wilm, T. Kasponas, S. H. Aarnink, L. F. de Geus-Oei, F. Brochin, T. Martinez, A. Michailovas, W. Muller Mobold, J. Jaros, J. Veltman, B. Cox, S. Manohar, Fully three-dimensional sound speed-corrected multi-wavelength photoacoustic breast tomography. arXiv:2308.06754 [physics.med-ph] (2023).

[38] G. L. G. Menezes, R. M. Pijnappel, C. Meeuwis, R. Bisschops, J. Veltman, P. T. Lavin, M. J. van de Vijver, R. M. Mann, Downgrading of breast masses suspicious for cancer by using optoacoustic breast imaging. Radiology 288, 355–365 (2018). 29664342 10.1148/radiol.2018170500

[39] E. I. Neuschler, R. Butler, C. A. Young, L. D. Barke, M. L. Bertrand, M. Böhm-Vélez, S. Destounis, P. Donlan, S. R. Grobmyer, J. Katzen, K. A. Kist, P. T. Lavin, E. V. Makariou, T. M. Parris, K. J. Schilling, F. L. Tucker, B. E. Dogan, A pivotal study of optoacoustic imaging to diagnose benign and malignant breast masses: A new evaluation tool for radiologists. Radiology 287, 398–412 (2018). 29178816 10.1148/radiol.2017172228

[40] B. E. Dogan, G. L. G. Menezes, R. S. Butler, E. I. Neuschler, R. Aitchison, P. T. Lavin, F. L. Tucker, S. R. Grobmyer, P. M. Otto, A. T. Stavros, Optoacoustic imaging and gray-scale US features of breast cancers: Correlation with molecular subtypes. Radiology 292, 564–572 (2019). 31287388 10.1148/radiol.2019182071

[41] US Food and Drug Administration, Summary of safety and effectiveness data (ssed): Imagio® Breast Imaging System – P200003 (2021); www.fda.gov/medical-devices/recently-approved-devices/imagior-breast-imaging-system-p200003.

[42] M. Yang, L. Zhao, F. Yang, N. Su, C. Zhao, Y. Gui, Y. Wei, R. Zhang, J. Li, T. Han, X. He, L. Zhu, H. Wu, C. Li, Y. Jiang, Quantitative analysis of breast tumours aided by three-dimensional photoacoustic/ultrasound functional imaging. Sci. Rep. 10, 8047 (2020). 32415203 10.1038/s41598-020-64966-6 PMC7229157

[43] B. Huang, J. Xia, K. Maslov, L. V. Wang, Improving limited-view photoacoustic tomography with an acoustic reflector. J. Biomed. Opt. 18, 110505 (2013). 24285421 10.1117/1.JBO.18.11.110505 PMC3842504

[44] K. Maslov, G. Stoica, L. V. Wang, In vivo dark-field reflection-mode photoacoustic microscopy. Opt. Lett. 30, 625–627 (2005). 15791997 10.1364/ol.30.000625

[45] S. A. S. Karam, D. O’Loughlin, B. L. Oliveira, M. O'Halloran, B. M. Asl, Weighted delay-and-sum beamformer for breast cancer detection using microwave imaging. Measurement 177, 109283 (2021).

[46] S. Hakakzadeh, S. M. Mostafavi, Z. Kavehvash, “Unipolar back-projection algorithm for photoacoustic tomography,” in *2022 IEEE International Ultrasonics Symposium (IUS)* (IEEE, 2022), pp. 1–4.

[47] S. Tzoumas, A. Nunes, I. Olefir, S. Stangl, P. Symvoulidis, S. Glasl, C. Bayer, G. Multhoff, V. Ntziachristos, Eigenspectra optoacoustic tomography achieves quantitative blood oxygenation imaging deep in tissues. Nat. Commun. 7, 12121 (2016). 27358000 10.1038/ncomms12121 PMC4931322

[48] S. S. S. Choi, B. Lashkari, E. Dovlo, A. Mandelis, Wavelength-modulated differential photoacoustic radar imager (WM-DPARI): Accurate monitoring of absolute hemoglobin oxygen saturation. Biomed. Opt. Express 7, 2586–2596 (2016). 27446691 10.1364/BOE.7.002586 PMC4948615

[49] T. Zhao, A. E. Desjardins, S. Ourselin, T. Vercauteren, W. Xia, Minimally invasive photoacoustic imaging: Current status and future perspectives. Photoacoustics 16, 100146 (2019). 31871889 10.1016/j.pacs.2019.100146 PMC6909166

[50] Z. Fang, C. Wang, J. Yang, Z. Song, C. Xie, Y. Ji, Z. Wang, X. Du, Q. Zheng, C. Chen, Z. Hu, Y. Zhong, Oxyhaemoglobin saturation NIR-IIb imaging for assessing cancer metabolism and predicting the response to immunotherapy. Nat. Nanotechnol. 19, 124–130 (2024). 37696994 10.1038/s41565-023-01501-4

[51] Q. Zhu, A. Jr Ricci, P. Hegde, M. Kane, E. Cronin, A. Merkulov, Y. Xu, B. Tavakoli, S. Tannenbaum, Assessment of functional differences in malignant and benign breast lesions and improvement of diagnostic accuracy by using US-guided diffuse optical tomography in conjunction with conventional US. Radiology 280, 387–397 (2016). 26937708 10.1148/radiol.2016151097 PMC4976463

[52] W. A. Berg, L. Gutierrez, M. S. NessAiver, W. B. Carter, M. Bhargavan, R. S. Lewis, O. B. Ioffe, Diagnostic accuracy of mammography, clinical examination, US, and MR imaging in preoperative assessment of breast cancer. Radiology 233, 830–849 (2004). 15486214 10.1148/radiol.2333031484

[53] H. M. Langevin, J. A. Yandow, Relationship of acupuncture points and meridians to connective tissue planes. Anat. Rec. 269, 257–265 (2002). 12467083 10.1002/ar.10185

[54] A. C. Ahn, M. Park, J. R. Shaw, C. A. McManus, T. J. Kaptchuk, H. M. Langevin, Electrical impedance of acupuncture meridians: The relevance of subcutaneous collagenous bands. PLOS ONE 5, e11907 (2010). 20689594 10.1371/journal.pone.0011907 PMC2912845

[55] W.-B. Zhang, G.-J. Wang, K. Fuxe, Classic and modern meridian studies: A review of low hydraulic resistance channels along meridians and their relevance for therapeutic effects in traditional chinese medicine. Evid. Based Complement. Alternat. Med. 2015, 410979 (2015). 25821487 10.1155/2015/410979 PMC4363694

[56] *125 Questions: Exploration and Discovery* (Science/AAAS Custom Publishing Office, 2021).

[57] S. C. Cheng, C. H. Lin, Y. J. Chang, T. H. Lee, S. J. Ryu, C. H. Chen, H. K. Chang, C. J. Chang, W. L. Hu, Y. C. Hung, Fire-heat and Qi deficiency syndromes as predictors of short-term prognosis of acute ischemic stroke. J. Altern. Complement. Med. 19, 721–728 (2013). 23600945 10.1089/acm.2012.0546 PMC3731676

[58] S. Schröder, J. Liepert, A. Remppis, J. H. Greten, Acupuncture treatment improves nerve conduction in peripheral neuropathy. Eur. J. Neurol. 14, 276–281 (2007). 17355547 10.1111/j.1468-1331.2006.01632.x

[59] Q. Ji, Y. Q. Luo, W. H. Wang, X. Liu, Q. Li, S. B. Su, Research advances in traditional Chinese medicine syndromes in cancer patients. J. Integr. Med. 14, 12–21 (2016). 26778224 10.1016/S2095-4964(16)60237-6

[60] P. Hu, L. Li, L. V. Wang, Location-dependent spatiotemporal antialiasing in photoacoustic computed tomography. IEEE Trans. Med. Imaging 42, 1210–1224 (2023). 36449587 10.1109/TMI.2022.3225565 PMC10171137

[61] “American national standard for the safe use of lasers” (ANSI Z136.1–2022, Laser Institute of America, 2022).

[62] L. V. Wang, H. I. Wu, *Biomedical Optics: Principles and Imaging* (John Wiley & Sons Press, 2007).

[63] R. G. Barr, A. Rim, R. Graham, W. Berg, J. R. Grajo, Speed of sound imaging improved image quality in breast sonography. Ultrasound Q. 25, 141–144 (2009). 19730076 10.1097/RUQ.0b013e3181b789aa

[64] J. A. Jensen, S. I. Nikolov, K. L. Gammelmark, M. H. Pedersen, Synthetic aperture ultrasound imaging. Ultrasonics 44, E5–E15 (2006). 16959281 10.1016/j.ultras.2006.07.017

[65] V. Perrot, M. Polichetti, F. Varray, D. Garcia, So you think you can DAS? A viewpoint on delay-and-sum beamforming. Ultrasonics 111, 106309 (2021). 33360053 10.1016/j.ultras.2020.106309

[66] S. Arridge, P. Beard, M. Betcke, B. Cox, N. Huynh, F. Lucka, O. Ogunlade, E. Zhang, Accelerated high-resolution photoacoustic tomography via compressed sensing. Phys. Med. Biol. 61, 8908–8940 (2016). 27910824 10.1088/1361-6560/61/24/8908

[67] K. B. Chowdhury, J. Prakash, A. Karlas, D. Justel, V. Ntziachristos, A synthetic total impulse response characterization method for correction of hand-held optoacoustic images. IEEE Trans. Med. Imaging 39, 3218–3230 (2020). 32324545 10.1109/TMI.2020.2989236

[68] N. Awasthi, G. Jain, S. K. Kalva, M. Pramanik, P. K. Yalavarthy, Deep neural network-based sinogram super-resolution and bandwidth enhancement for limited-data photoacoustic tomography. IEEE Trans. Ultrason. Ferroelectr. Freq. Control 67, 2660–2673 (2020). 32142429 10.1109/TUFFC.2020.2977210

[69] H. Shan, G. Wang, Y. Yang, Accelerated correction of reflection artifacts by deep neural networks in photo-acoustic tomography. Appl. Sci. 9, 2615 (2019).

[70] N. Davoudi, X. L. Dean-Ben, D. Razansky, Deep learning optoacoustic tomography with sparse data. Nat. Mach. Intell. 1, 453–460 (2019).

[71] L. Li, L. Zhu, C. Ma, L. Lin, J. Yao, L. Wang, K. Maslov, R. Zhang, W. Chen, J. Shi, L. V. Wang, Single-impulse panoramic photoacoustic computed tomography of small-animal whole-body dynamics at high spatiotemporal resolution. Nat. Biomed. Eng. 1, 0071 (2017). 29333331 10.1038/s41551-017-0071 PMC5766044

[72] K. Zuiderveld, “Contrast limited adaptive histogram equalization,” in *Graphics Gems IV* (Academic Press Professional Inc., 1994), pp. 474–485.

[73] J. Kim, J. K. Lee, K. M. Lee, “Accurate image super-resolution using very deep convolutional networks,” in *2016 IEEE Conference on Computer Vision and Pattern Recognition (CVPR)* (IEEE, 2016), pp. 1646–1654.

[74] S. Ghavami, M. Bayat, M. Fatemi, A. Alizad, Quantification of morphological features in non-contrast-enhanced ultrasound microvasculature imaging. IEEE Access 8, 18925–18937 (2020). 32328394 10.1109/ACCESS.2020.2968292 PMC7179329

[75] R. Ternifi, Y. Wang, J. Gu, E. C. Polley, J. M. Carter, S. Pruthi, J. C. Boughey, R. T. Fazzio, M. Fatemi, A. Alizad, Ultrasound high-definition microvasculature imaging with novel quantitative biomarkers improves breast cancer detection accuracy. Eur. Radiol. 32, 7448–7462 (2022). 35486168 10.1007/s00330-022-08815-2 PMC9616967

[76] *BI-RADS®: ACR Breast Imaging Reporting and Data System* (American College of Radiology, 2013).

[77] W. Gomez-Flores, J. Hernandez-Lopez, Assessment of the invariance and discriminant power of morphological features under geometric transformations for breast tumor classification. Comput. Methods Programs Biomed. 185, 105173 (2020). 31710986 10.1016/j.cmpb.2019.105173

[78] B. Karimi, A. Krzyzak, A novel technique for detecting suspicious lesions in breast ultrasound images. Concurr. Comput. Pract. Exper. 28, 2237–2260 (2016).

[79] S. Raza, A. L. Goldkamp, S. A. Chikarmane, R. L. Birdwell, US of breast masses categorized as BI-RADS 3, 4, and 5: Pictorial review of factors influencing clinical management. Radiographics 30, 1199–1213 (2010). 20833845 10.1148/rg.305095144

[80] A. Gronningsaeter, B. A. J. Angelsen, A. Heimdal, H. G. Torp, Vessel wall detection and blood noise reduction in intravascular ultrasound imaging. IEEE Trans. Ultrason. Ferroelectr. Freq. Control 43, 359–369 (1996).

[81] A. Altmann, L. Tolosi, O. Sander, T. Lengauer, Permutation importance: A corrected feature importance measure. Bioinformatics 26, 1340–1347 (2010). 20385727 10.1093/bioinformatics/btq134

[82] “Near-infrared window in biological tissue”; https://en.wikipedia.org/wiki/Near-infrared_window_in_biological_tissue#cite_note-10.

[83] J. G. Koelzer, G. Mitic, J. Otto, W. Zinth, “Measurements of the optical properties of breast tissue using time-resolved transillumination,” in *Proceedings Volume 2326, Photon Transport in Highly Scattering Tissue*, Lille, France, 31 January 1995 (SPIE, 1995); 10.1117/12.200841.

[84] S. A. Ermilov, T. Khamapirad, A. Conjusteau, M. H. Leonard, R. Lacewell, K. Mehta, T. Miller, A. A. Oraevsky, Laser optoacoustic imaging system for detection of breast cancer. J. Biomed. Opt. 14, 024007 (2009). 19405737 10.1117/1.3086616

[85] D. Garcia, “Make the most of MUST, an open-source Matlab Ultrasound Toolbox,” *2021 IEEE International Ultrasonics Symposium (IUS)* (IEEE, 2021), pp. 1–4.

[86] D. D. Royston, R. S. Poston, S. A. Prahl, Optical properties of scattering and absorbing materials used in the development of optical phantoms at 1064 nm. J. Biomed. Opt. 1, 110–116 (1996). 23014651 10.1117/12.227698

[87] R. Michels, F. Foschum, A. Kienle, Optical properties of fat emulsions. Opt. Express 16, 5907–5925 (2008). 18542702 10.1364/oe.16.005907

[88] T. Hopp, A. Stromboni, N. Duric, N. V. Ruiter, “Evaluation of breast tissue characterization by ultrasound computer tomography using a 2D/3D image registration with mammograms,” in *2013 IEEE International Ultrasonics Symposium (IUS)* (IEEE, 2013), pp. 647–650.

[89] A. Jozefczak, K. Kaczmarek, M. Kubovcikova, Z. Rozynek, T. Hornowski, The effect of magnetic nanoparticles on the acoustic properties of tissue-mimicking agar-gel phantoms. J. Magn. Magn. Mater. 431, 172–175 (2017).

