# Supplementary material for: High-speed photoacoustic and ultrasonic computed tomography of the breast tumor for early diagnosis with enhanced accuracy
Source: Sci Adv. 2025 Oct 8;11(41):eadz2046. doi: 10.1126/sciadv.adz2046 (PMC12506975; doi:10.1126/sciadv.adz2046)
Supplement: Supplementary file 1 — Figs. S1 to S18 Tables S1 to S5 Legends for movies S1 to S4 Pseudocode References [file sciadv.adz2046_sm.pdf]

Science Advances
AAAS

Supplementary Materials for

High-speed photoacoustic and ultrasonic computed tomography of the breast tumor for early diagnosis with enhanced accuracy

Keer Huang *et al.*

Corresponding author: Li Lin, linliokok@zju.edu.cn

Sci. Adv. **11**, eadz2046 (2025)
DOI: 10.1126/sciadv.adz2046

The PDF file includes:

Figs. S1 to S18
Tables S1 to S5
Legends for movies S1 to S4
Pseudocode
References

Other Supplementary Material for this manuscript includes the following:

Movies S1 to S4

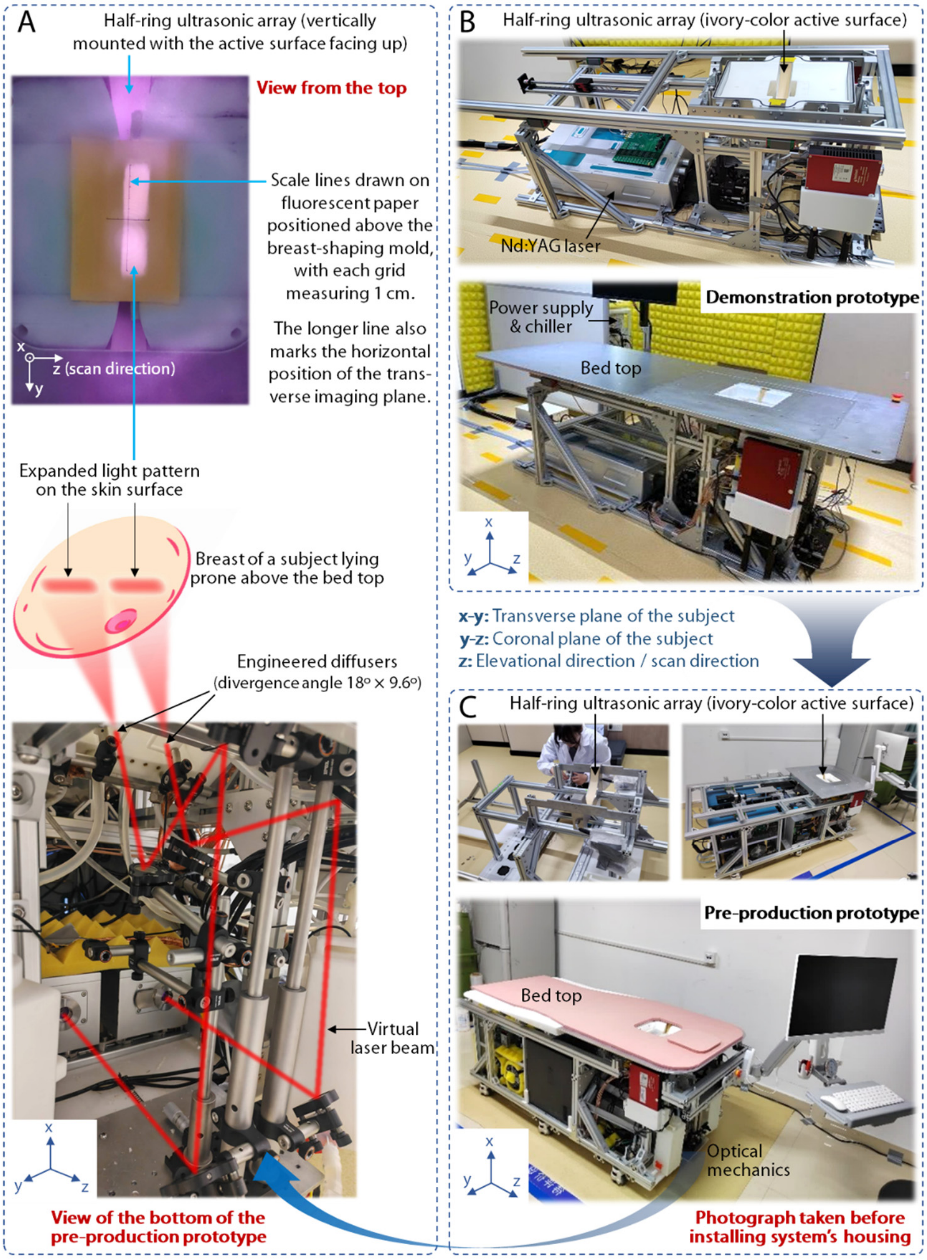

Fig. S1.

Photographs of two HDMI prototypes built from scratch. (**A**) Expanded light pattern on fluorescent paper near the skin surface altitude (top) and virtual light paths within the system (bottom). (**B**) Demonstration prototype. The laser's power supply and chiller, with large footprints, are placed behind the white divider. (**C**) Pre-production prototype with the system enclosure removed, showing all modules, including the lasers' power supplies and chillers, located beneath the imaging platform.

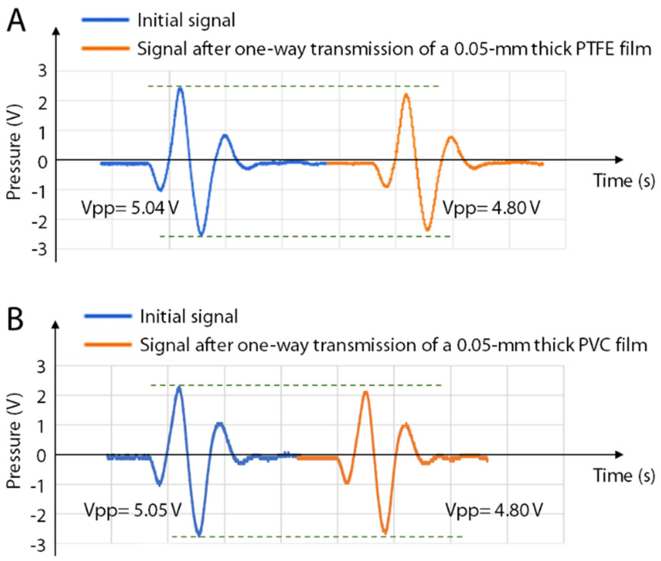

Fig. S2.

Measurement of ultrasound attenuation after one-way transmission through PTFE (A) and PVC films (B). The signal was transmitted from a single-element transducer with a 5-MHz central frequency and received by another transducer with a 2.25-MHz central frequency positioned opposite. To simulate acoustic signal attenuation around 3 MHz, we filtered the received signals within the 2–4 MHz range, both with and without PTFE or PVC films positioned between the transducers. During the clinical study, researchers refined the setup by replacing PTFE with PVC to reduce breast deformation caused by shear forces from scanning friction.

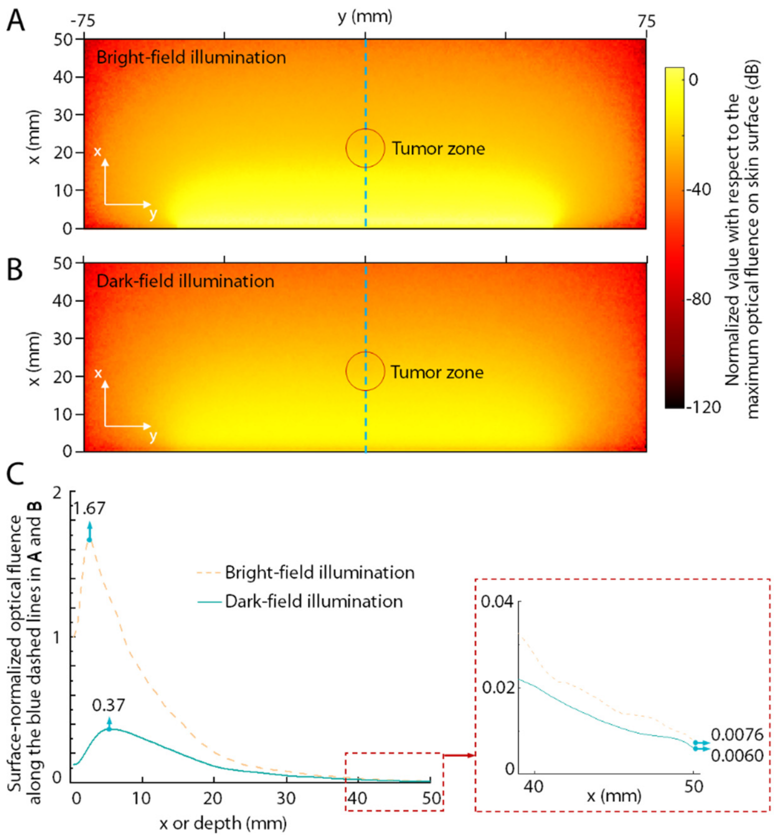

Fig. S3.

Monte Carlo simulation of optical fluence in breast tissue (μ_a = 0.04 cm^{-1}; μ_s' = 6.4 cm^{-1} at 1064 nm) (*82-84*) **on the transverse imaging plane.** The model incorporates a tumor (μ_a = 0.1 cm^{-1}; μ_s' = 10.0 cm^{-1}) (*82, 84*) centered at a depth of 2 cm. The optical fluence distributions for both bright-field (**A**) and dark-field (**B**) illumination strategies are presented. (**C**) Bright-field surface-normalized optical attenuation curves along the blue dashed lines in (A) and (B). While bright-field illumination offers higher overall efficiency, its optical fluence variation with depth is substantially larger than that of dark-field illumination (220-fold vs. 62-fold), imposing greater demands on the dynamic range of data acquisition systems.

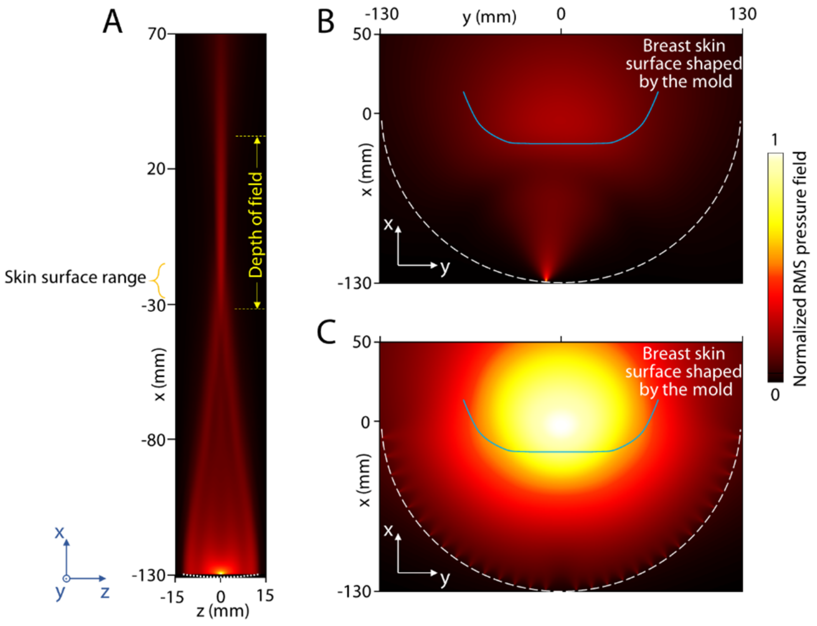

Fig. S4.

Simulated ultrasound transmit beam profiles using the MATLAB UltraSound Toolbox (*85*). (**A**) Acoustic diffraction field on the x-z plane, illustrating the acoustic focus that defines the snapshot imaging cross-section. (**B**) Acoustic diffraction field of a single transmit element on the transverse (x-y) plane. (**C**) Summed acoustic diffraction field of 30 transmit elements evenly distributed across the array (marked by the white dashed line). RMS, Root mean square.

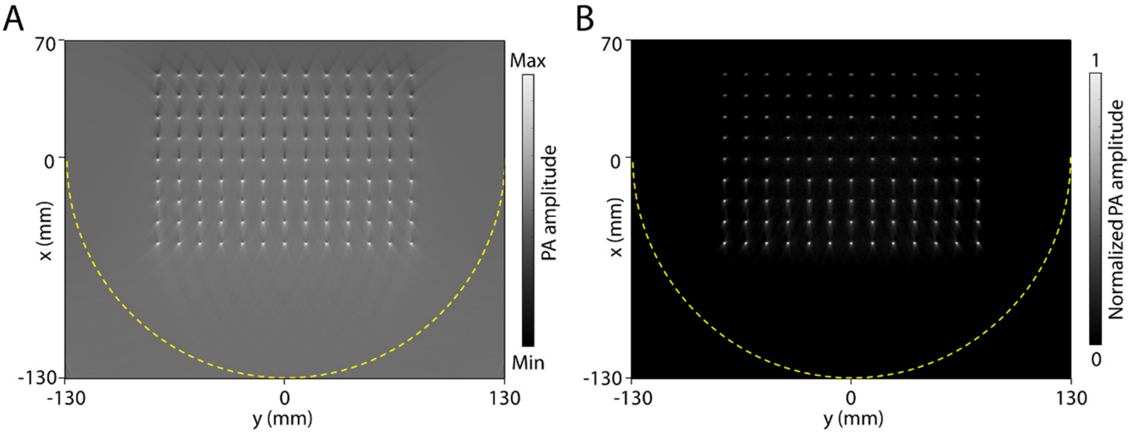

Fig. S5.

Numerical simulations for PACT reconstruction in the transverse plane. (**A**) PA targets reconstructed using traditional DAS reconstruction algorithms, displaying bipolar image features with both positive and negative amplitudes. (**B**) The same PA targets reconstructed using the unipolar weighted DAS method (*45, 46*), resulting in unipolar positive image features. Negative artifacts present in the bipolar reconstruction are effectively suppressed in the unipolar counterpart. The ultrasonic array is indicated by the yellow dashed line.

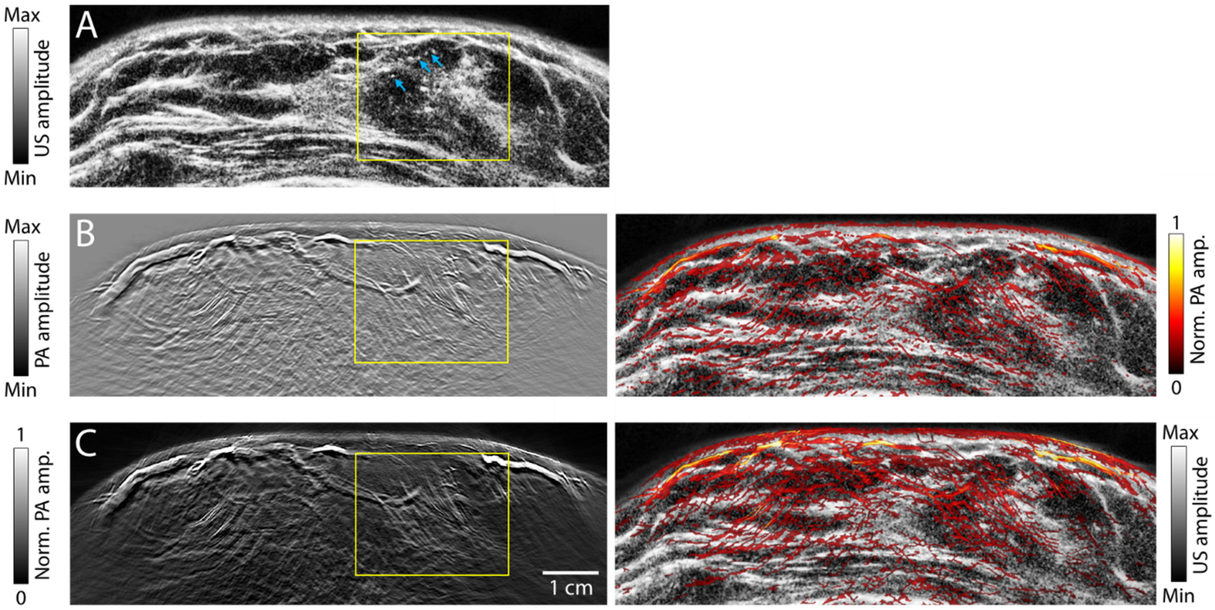

Fig. S6.

Dual-modal HDMI of breast invasive carcinoma (enclosed in yellow boxes) on the transverse plane. (**A**) URCT image of the mass, revealing associated microcalcifications marked by blue arrows. (**B**) PACT image reconstructed using DAS bipolar reconstruction algorithms (left) and its fused version, where the PACT angiogram is overlaid on the URCT anatomy (right). Some blood vessels appear discontinuous in the fused image due to thresholding of negative pixels in the bipolar data. (**C**) The same PACT data reconstructed using the unipolar weighted DAS method (*45, 46*). Artifacts are less prominent, and blood vessels appear more continuous in the fused image, as all vessels are represented with positive amplitudes in the unipolar reconstruction.

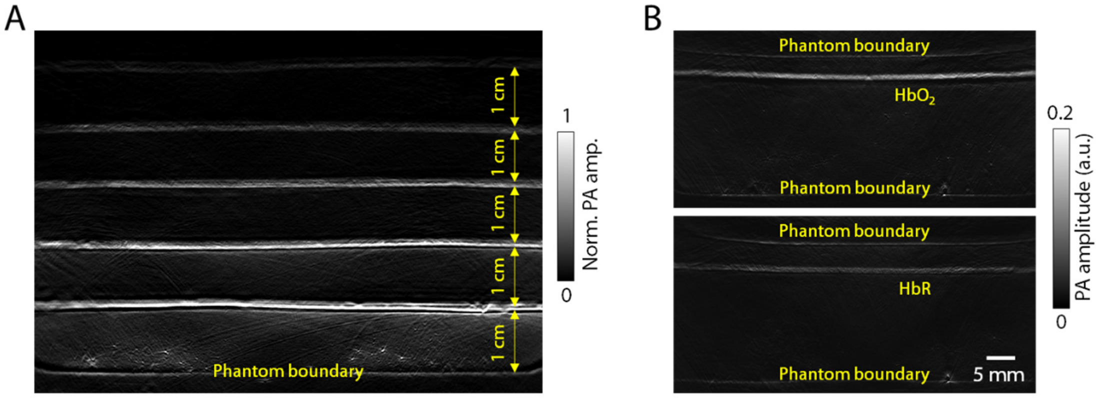

Fig. S7.

HDMI of blood in breast-mimicking phantoms. (**A**) Imaging depth validation of HDMI, which revealed five tunnels filled with oxidized blood at different depths in an agar brick. The agar brick was mixed with **0.10**% Indian ink (*86*) and **4.6**% intralipid (**20**%) (*87*), mimicking breast tissue absorption and scattering coefficients (μ_a = **0.04** cm^{-1}; $\mu_s{}'$ = **6.4** cm^{-1} at **1064** nm) (*82-84*). (**B**) PACT images of a phantom tunnel filled with either oxidized (top) or deoxidized blood (bottom), showing higher signal amplitude from the oxidized blood.

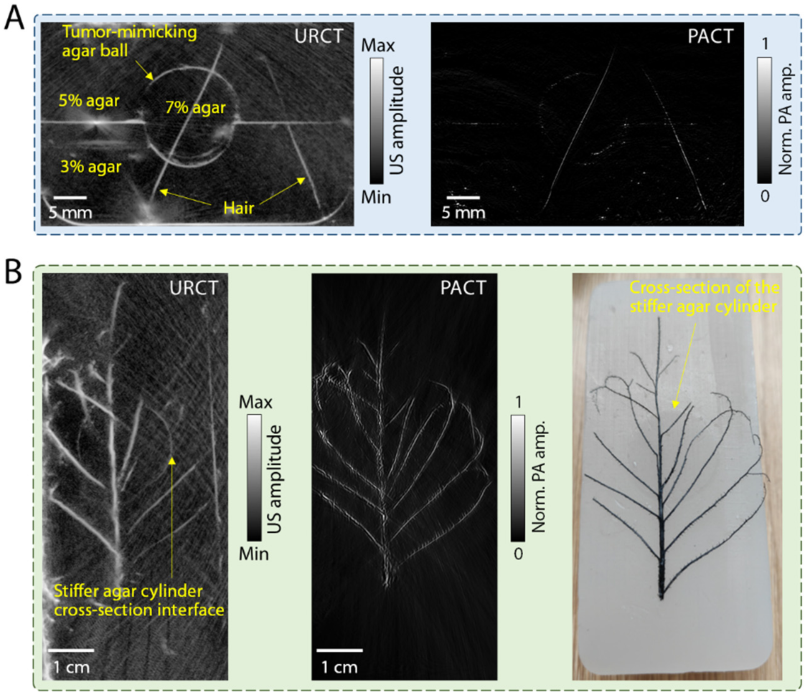

Fig. S8.

HDMI of optical absorbers embedded in acoustically heterogeneous phantoms. These results suggest that moderate acoustic heterogeneity (e.g., a tumor in breast tissue) may slightly deform PACT image features, but does not introduce new ones. (**A**) URCT and PACT images of two human black hairs embedded in an agar phantom (**3**% and **5**% agar for adipose and glandular tissues, respectively), with a **7**% agar ball placed in the middle to represent a tumor with a faster speed of sound (*88, 89*). (**B**) URCT, PACT, and a photograph of a leaf skeleton phantom, which was sandwiched between two agar bricks. These bricks were created by cutting an agar phantom (**3**% agar) in half, with a stiffer agar cylinder (**7**% agar) originally embedded in the center. The photograph was taken before the two agar bricks were glued together.

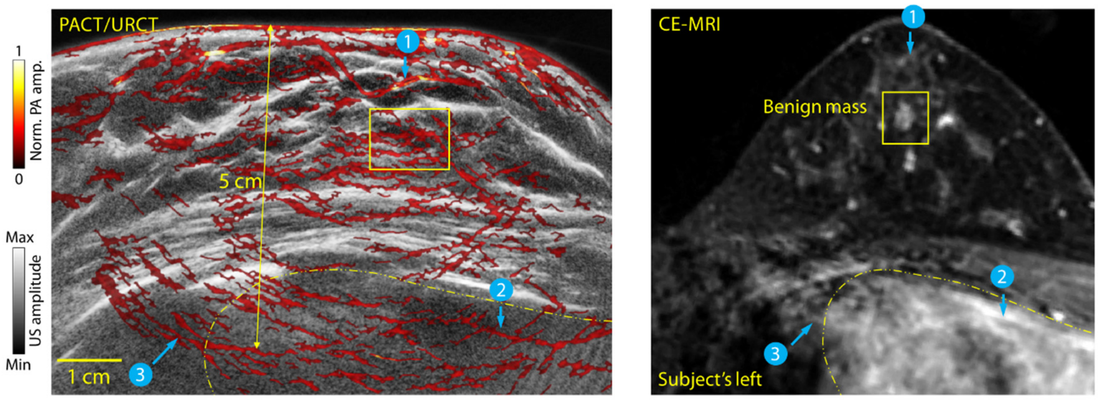

Fig. S9.

Comparison of HDMI and CE-MRI transverse frames from a patient with a benign mass. Feature concordance is observed in certain vessels (indicated by blue arrows) within the breast and beyond the chest wall between PACT and CE-MRI. Blood vessels in the PACT images were identified based on their tubular structures with either branching or sinuous morphologies. The yellow dashed lines delineate the heart region, which appears as a whitish, cloudy area in both the URCT and CE-MRI images.

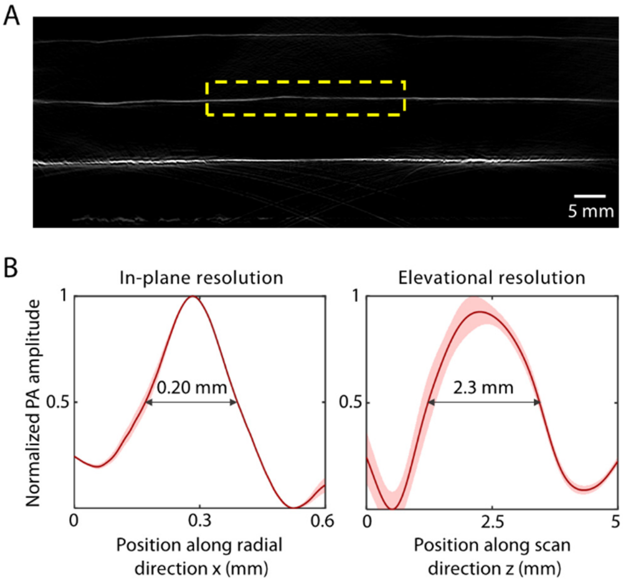

Fig. S10.

Spatial resolution measurement of HDMI. (**A**) PACT images of three tungsten wires (0.04 mm diameter) embedded in a tissue-mimic phantom. (**B**) Line spread plots of the wire section within the yellow dashed box, showing 0.2 mm in-plane resolution and 2.3 mm elevational resolution. The in-plane resolution is determined by measuring the full-width at half-maximum (FWHM) of the line spread function of the wire. The elevational resolution is quantified based on the FWHM of the line profile along the z-direction after scanning the tungsten wire over a 1 cm range in 100-µm steps.

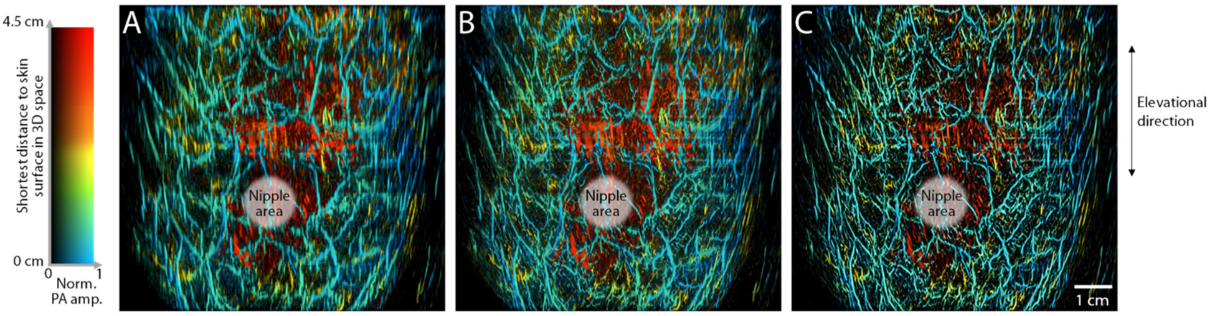

Fig. S11.

PACT maximum amplitude projection (MAP) on the coronal plane. (**A**) MAP image after applying adaptive depth compensation to the raw image. (**B**) Processed image with enhanced elevational sharpness obtained using the deep convolutional network. (**C**) Final image after refinement with the Hessian-based Frangi filter.

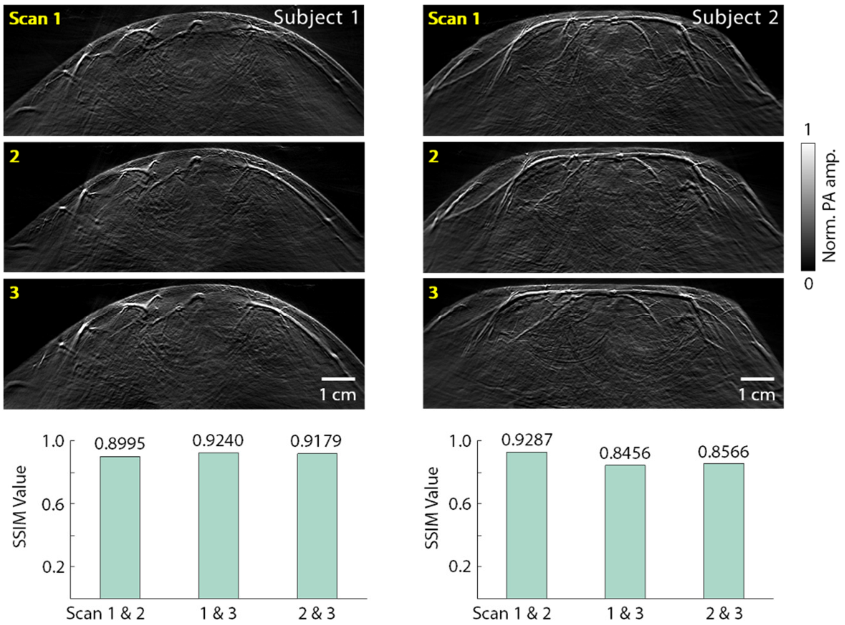

Fig. S12.

Representative PACT images of similar breast cross-sections demonstrating reproducibility, assessed by the structural similarity (SSIM) value between the two images. The SSIM metrics are calculated after removing a 3-mm thick layer from the skin surface to exclude the contribution from skin structures.

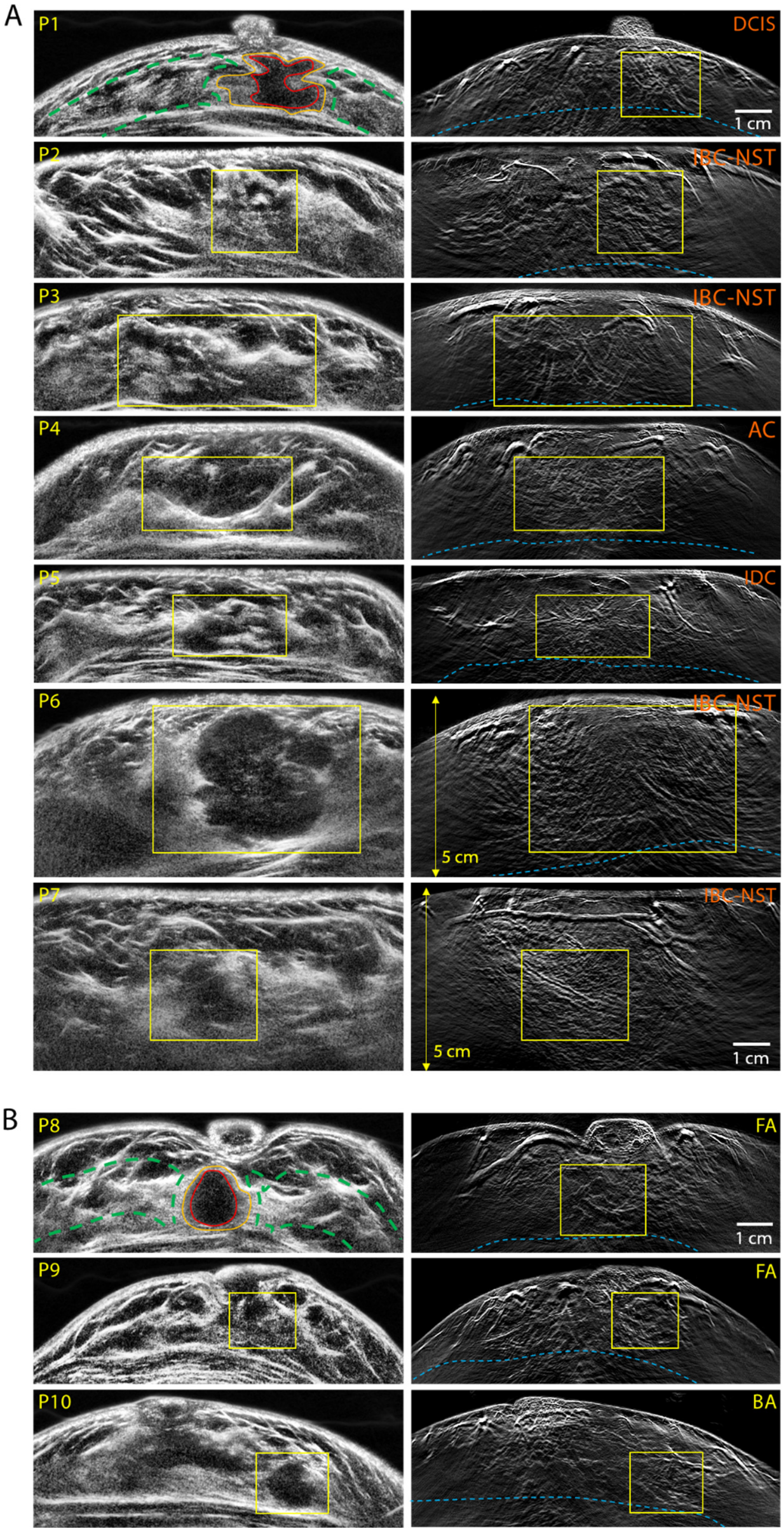

A
P1
DCIS
1 cm
P2
IBC-NST
P3
IBC-NST
P4
AC
P5
IDC
P6
IBC-NST
5 cm
P7
IBC-NST
5 cm
1 cm
B
P8
FA
1 cm
P9
FA
P10
BA

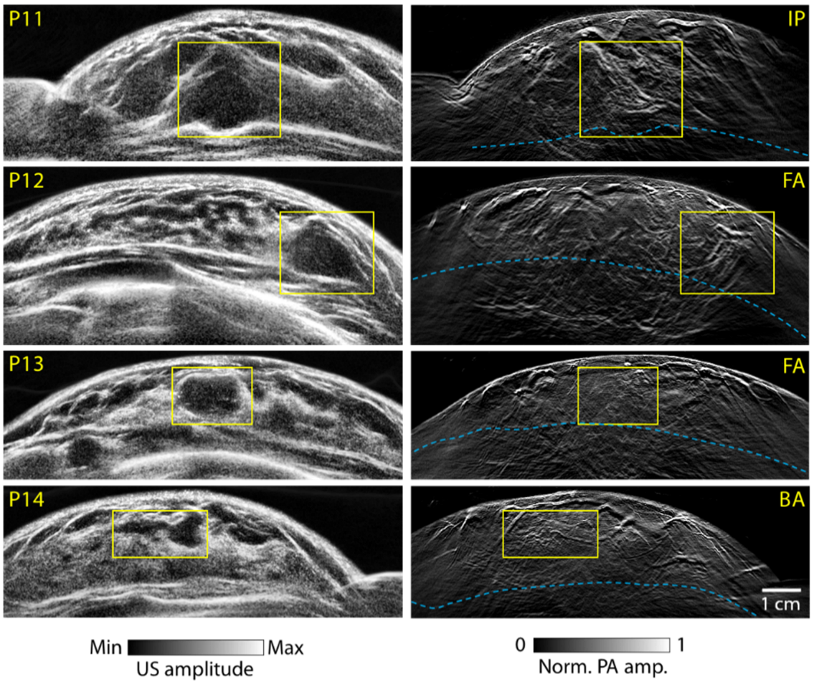

Fig. S13.

Additional HDMI dual-modal images of patients with breast malignant (A) and benign masses (B) on the transverse plane. The tumor zones, boundary zones, and reference zones determined by the URCT images are delineated in P1 and P8 with red lines, orange lines, and green dashed lines, respectively. Tumor regions are enclosed with yellow boxes, and the chest walls recognizable in URCT images are outlined using blue dashed lines in the PACT counterpart. The images are cropped either beyond the chest wall in small/moderate breasts or at 5 cm in relatively large breasts. This approach ensures that we capture the relevant depth information while excluding distorted features from ribs. AC, Apocrine carcinoma; BA, Breast adenosis; DCIS, Ductal carcinoma *in situ*; FA, Fibroadenoma; IBC-NST, Invasive breast carcinoma of no special type; IDC, Invasive ductal carcinoma; IP, Intraductal papilloma.

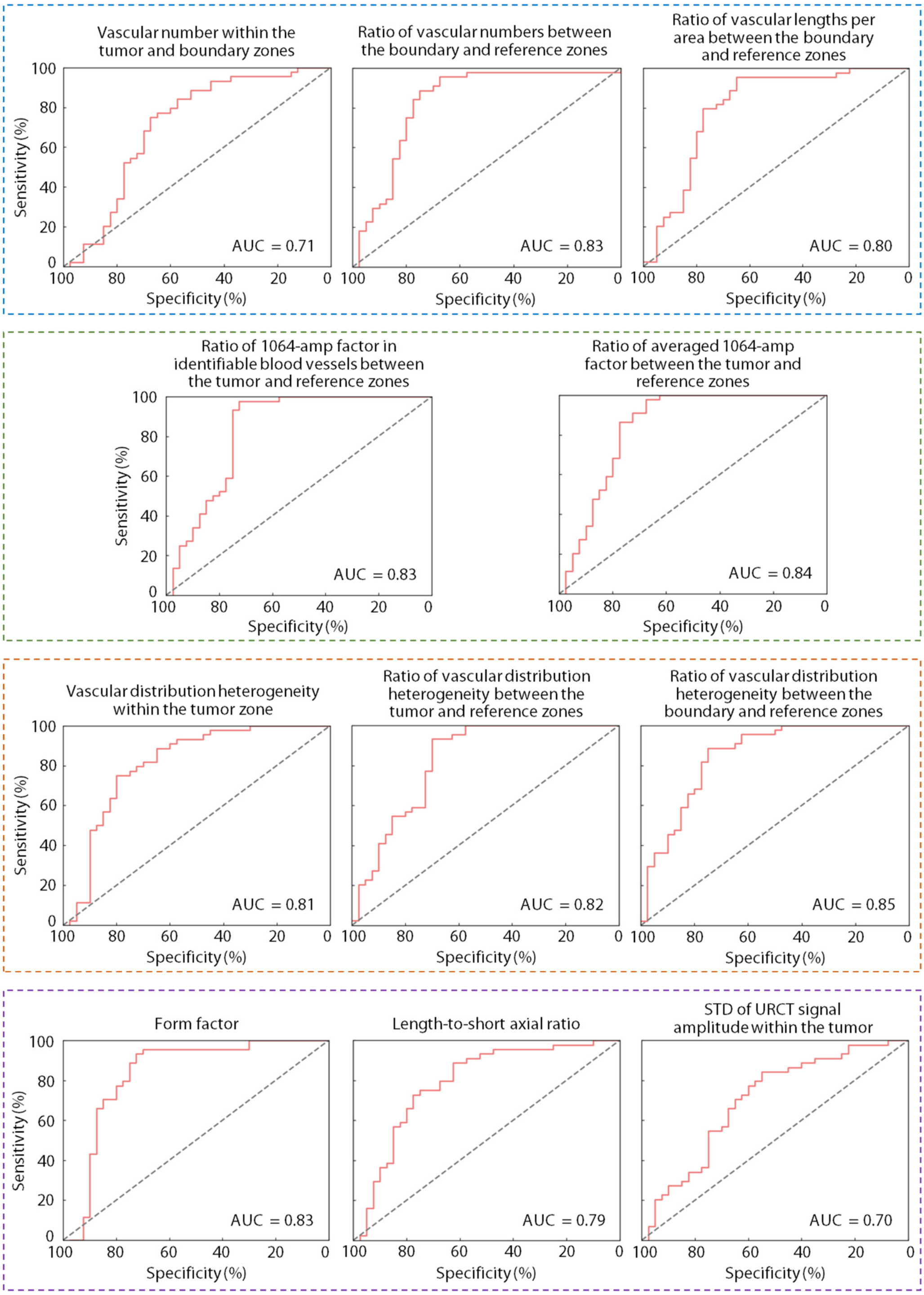

Fig. S14.

ROC curves of the diagnostic model after randomly permuting each of the 11 features in the model. The importance of each image feature in the model can be assessed based on the changes in the area under the ROC curve (AUC) before and after the random permutation. The ROC curves are presented in the same order as shown in (Figs. 5A to D).

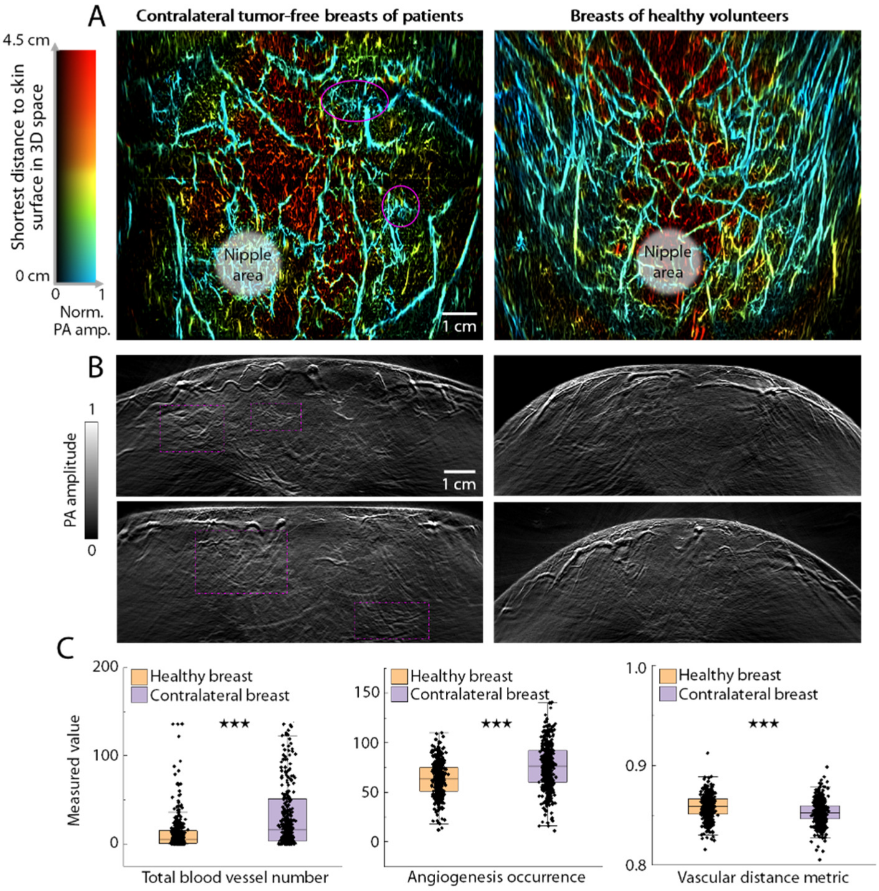

Fig. S15.

Angiogenesis findings in contralateral tumor-free breasts of patients. (**A**) PACT images of the breast vasculature with color-encoded depths in a contralateral tumor-free breast (left) and a healthy breast (right). Regions with prolific vasculature are marked by magenta circles. (**B**) PACT images of contralateral tumor-free breasts of two patients on the transverse plane (left), showing more proliferative blood vessels than those in healthy subjects (right). (**C**) Error-bar plots differentiating contralateral breasts and healthy breasts using PACT features related to blood vessels. ***$P < 0.001$. Healthy breasts (n = 32 breasts), contralateral breasts (n = 40 breasts).

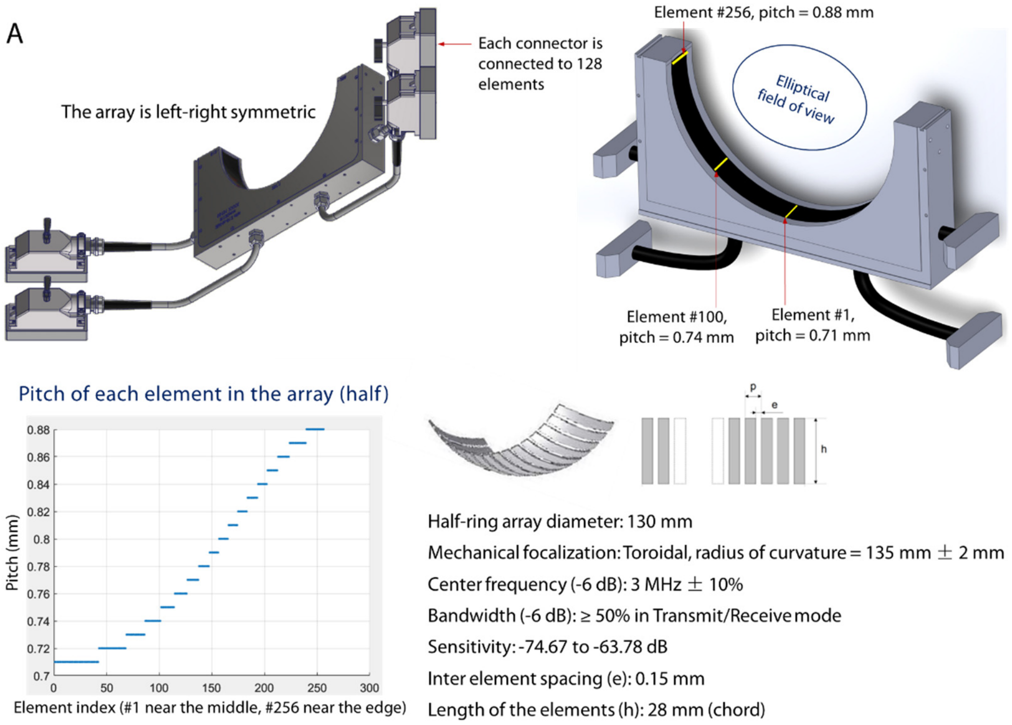

B

Input parameters			Output parameters			
Frequency	3	MHz	Wavelength		0.49	mm
Active diameter	28	mm	Near Field		397.3	mm
Flat active area (yes/no)	☐		Focal distance		120.15	mm
Radius of curvature	135	mm	Beam width (-6dB)		2.28	mm
			Field depth (-6dB)	from	97.2	mm
Medium velocity	1480	m/s		to	162.27	mm

Fig. S16.

Design details and key acoustic properties of the 3-MHz transducer array. (**A**) Manufacturing design sketch of the array viewed from the bottom (top left). The illustrative diagram shows the element orientations and the elliptical field of view (top right). The pitch distribution and specifications of the transducer elements, which are symmetric about the left–right axis (bottom). (**B**) Simulated parameters of the acoustic diffraction field based on the array specifications (https://www.imasonic.com/industry/online-design/).

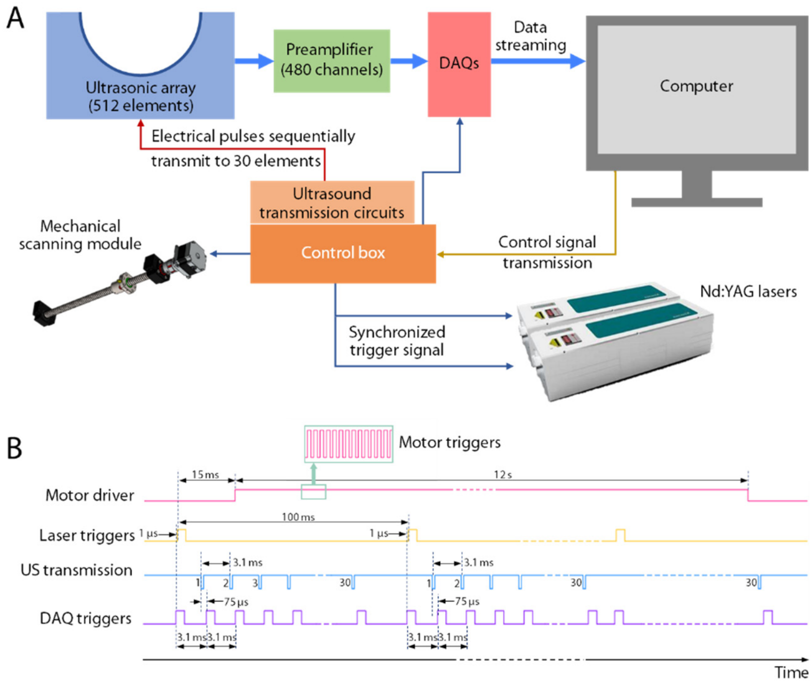

Fig. S17.

Signal flow (A) and timing diagrams (B) of HDMI pulse transmission and signal acquisition sequence. The motor driver was synchronized with continuous triggers repeated at 4.8 kHz lasting for 12 seconds, and began scanning the bed 15 ms after the first laser/DAQ triggers.

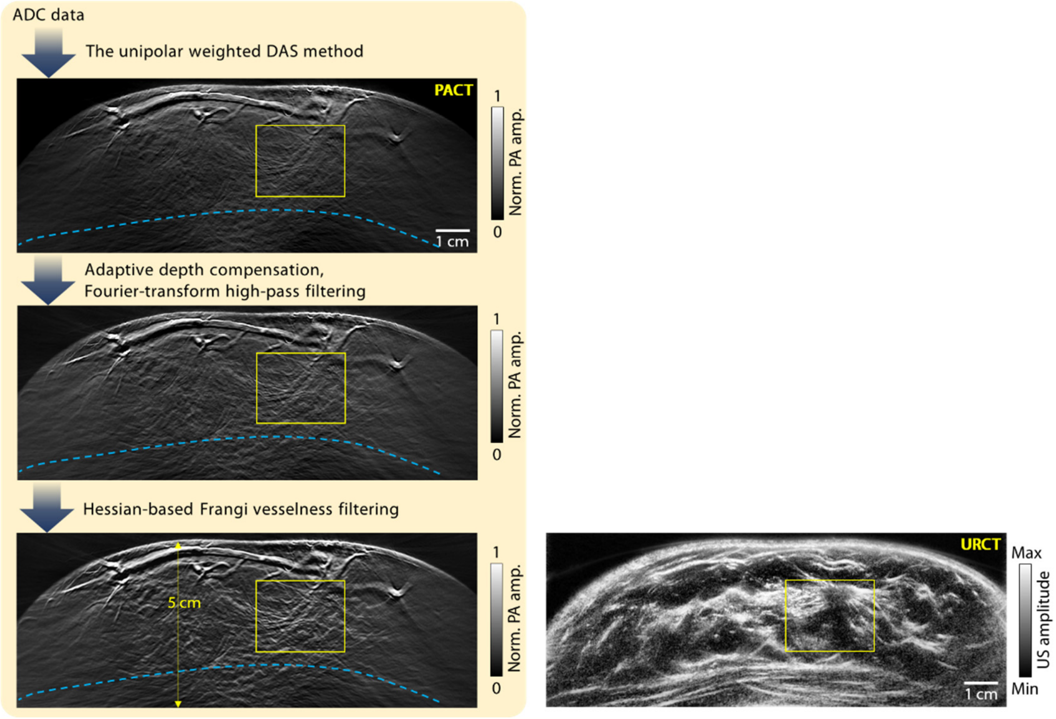

Fig. S18.

HDMI of a breast with invasive carcinoma (grade 3) on the transverse plane. The three PACT images on the left illustrate the workflow and effects of post-processing steps. Tumor regions are enclosed by yellow boxes, and the chest walls are outlined with blue dashed lines. URCT counterpart of the same cross-section is shown at the bottom right.

Table S1.
Patient population in the study.

**Patient population**	**No. of subjects**	**No. of masses**
Total enrolled	170 (100%)	186 (100%)
**Population excluded from statistical analysis**		
Demonstration prototype validation	12 (7.1%)	12 (6.5%)
Pre-production prototype optimization	5 (2.9%)	7 (3.8%)
Tumor sizes beyond the field of view in depth	5 (2.9%)	5 (2.7%)
BI-RADS 4a masses with no biopsy record	1 (0.6%)	1 (0.5%)
**“Ground truth” population for statistical analysis**		
Training group	70(41.2%)	77 (41.4%)
Testing group	77 (45.3%)	84 (45.2%)

Table S2.

Image features quantified in PACT/URCT images.

Tumor features	Definition and quantification strategy
Ratio of vascular numbers between the boundary and reference zones	Vascular number: number of vascular segments in the skeleton map
Vascular number within the tumor and boundary zones	/
Ratio of vascular lengths per area between the boundary and reference zones	Vascular length: total length of vascular segments in the skeleton map
Vascular distribution heterogeneity within the tumor zone	Vascular distribution heterogeneity: standard deviation of vascular signal amplitude
Ratio of vascular distribution heterogeneity between the tumor and reference zones	/
Ratio of vascular distribution heterogeneity between the boundary and reference zones	/
Ratio of averaged 1064-amp factor between the tumor and reference zones	1064-amp factor: PA signal amplitude acquired with 1064-nm illumination
Ratio of averaged 1064-amp factor in identifiable blood vessels between the tumor and reference zones	1064-amp factor in blood vessels: PA signal amplitude acquired with 1064-nm illumination in the area occupied by segmented blood vessels
Form factor	$\text{Form factor} = \frac{4\pi \times \text{Tumor area}}{\text{Tumor perimeter}^2}$
Length-to-short axial ratio	Length ratio of the tumor's major axis to its minor axis
STD of URCT signal amplitude within the tumor	/

Table S3.
Summary of histopathologic findings.

Mass type at histopathologic examination	No. of masses
**HDMI training population**	
Malignant (53.2%, 41/77)	41 (100%)
IBC-NST	
Grade 1	1 (2.4%)
Grade 2	14 (34.1%)
Grade 3	20 (48.8%)
Grade not recorded	1 (2.4%)
Other malignant*	5 (12.2%)
Benign (46.8%, 36/77)	36 (100%)
FA	13 (36.1%)
IP	4 (11.1%)
BA	4 (11.1%)
HMG	3 (8.3%)
Mastitis	3 (8.3%)
DD	2 (5.6%)
Cyst	1 (2.8%)
BI-RADS 3 after follow-up over 6 months	6 (16.7%)
**HDMI testing population**	
Malignant (52.4%, 44/84)	44 (100%)
High-grade DCIS	3 (6.8%)
High-grade ADCIS	2 (4.5%)
IBC-NST	
Grade 1	1 (2.3%)
Grade 2	14 (31.8%)
Grade 3	17 (38.6%)
Grade not recorded	2 (4.5%)
Other malignant†	5 (11.4%)
Benign (47.6%, 40/84)	40 (100%)
FA	17 (42.5%)

IP	4 (10%)
BA	12 (30%)
HMG	5 (12.5%)
Other benign‡	2 (5%)

Note: ADCIS, Apocrine ductal carcinoma *in situ*; BA, Breast adenosis; DCIS, Ductal carcinoma *in situ*; DD, Breast duct dilatation; FA, Fibroadenoma; HMG, Hyperplasia of the mammary glands; IBC-NST, Invasive breast carcinoma of no special type; IP, Intraductal papilloma.

* Other malignant includes (5, 12.2% of malignant masses in training population): Encapsulated papillary carcinoma (1, 2.4%), high-grade ductal carcinoma *in situ* (2, 4.9%), low-grade ductal carcinoma *in situ* (1, 2.4%), lobular carcinoma *in situ* (1, 2.4%).

† Other malignant includes (5, 11.4% of malignant masses in testing population): Encapsulated papillary carcinoma (1, 2.3%), neuroendocrine carcinoma of the breast (1, 2.3%), invasive micropapillary carcinoma (1, 2.3%), intermediate grade ductal carcinoma *in situ* (1, 2.3%), invasive apocrine carcinoma (1, 2.3%).

‡ Other benign includes (2, 5% of benign masses in training population): Mastitis (1, 2.5%), phyllode tumor of the breast (1, 2.5%).

Table S4.

BI-RADS categories classified by clinical ultrasonography in the testing population (*n* = 84).

BI-RADS category from clinical ultrasound	No. of masses
Malignant (52.4%, 44/84)	44 (100%)
4a	2 (4.5%)
4b	19 (43.2%)
4c	11 (25%)
5	9 (20.1%)
6	3 (6.8%)
Benign (47.6%, 40/84)	40 (100%)
3	9 (22.5%)
4a	21 (52.5%)
4b	8 (20%)
4c	1 (2.5%)
4 (grade not recorded)	1 (2.5%)

Table S5.

Subgroup analysis by age, maximum tumor size, and center depth showed no significant differences in both sensitivity and specificity. We divided the subjects into three age groups: <40 years, 40–60 years, and >60 years; For mass size, tumors were grouped by: long axis <10 mm, 10–20 mm, 20–40 mm, and >40 mm; For center depth, tumors were divided into two groups: center depth <20 mm and ≥20 mm**.**

**Subgroup**	**Sensitivity**	**Specificity**
	Overall P Value	Overall P Value
Age group	0.820	0.918
Mass size	0.948	0.781
Center depth	0.705	0.878

Movie S1.

Video recorded in the clinic during HDMI scanning of a patient, demonstrating real-time dual-modal image reconstruction and display. Tumor-containing cross-sections show up in the real-time reconstruction window at ~12 seconds in the movie. Please note that the displayed images during patient scanning were reconstructed with bipolar features.

Movie S2.

Synchronized and coregistered PACT and URCT video recording at a 10-Hz frame rate of a healthy volunteer at a fixed breast cross-section, explicitly showing blood vessel vibrations at ~1 Hz within the breast tissue and extending beyond the chest wall. The subject held her breath for 10 seconds during this HDMI video recording.

Movie S3.

Volumetric PACT image of a B-cup breast with invasive micropapillary carcinoma (highlighted with a red box). The image is cropped beyond the interface between the breast and the chest wall.

Movie S4.

Volumetric PACT image of a D-cup breast with invasive carcinoma of no special type (highlighted with a red box). A penetration depth of 5 cm (shortest distance to the skin surface in 3D space) is presented.

Pseudocode for PACT image reconstruction and processing

1. Spatial interpolation

Virtual elements are generated between every two adjacent transducer array elements through linear interpolation, with their signals averaged from the adjacent elements.

2. Image reconstruction

Calculate the derivative signal (*45, 46*):

For (p=1 to all channels of the received signal) {
    Calculate the derivative of each channel signal.
    For the negative portion, multiply by a compensation factor that is linearly related to time.
    Multiply each channel signal by -1.
}

Precompute the Blackman window apodization weights based on element positions.

Initialize the first reconstructed image as an all-zero matrix.

For (n=1 to all elements in the virtual array) {
    For (m=1 to all pixels in the image) {
        Calculate the distance between the m_{th} pixel and the n_{th} element.
        Calculate the propagation time and obtain the delayed data from the original channel signal.
        Add the weighted delayed data to the pixel.
    }
}

Initialize the second reconstructed image as an all-zero matrix.

Repeat the delay-and-sum (DAS) steps to generate the second image using the derivative signal.

Normalize the first and second images respectively, add them together and set the negative values to zero to generate a unipolar image.

3. Image postprocessing

Implement depth-dependent compensation to ensure the brightness of deep vessels is comparable to that of superficial vessels.

Apply Fourier-transform high-pass filtering to suppress the low-frequency background noise.

Perform Hessian-based Frangi filtering for vascular network enhancement, followed by a weighted addition of the high-pass filtered image.

4. Volumetric image generation

Generate cross-sectional images using the above DAS method, and stack them along the scanning direction.

Perform iterative image registration between cross-sectional images in the transverse plane.

Choose the appropriate interpolation factor based on the scanning speed and apply linear interpolation along the scanning direction.

Use a deep convolutional neural network to process each layer of the image in the coronal plane (*73*).

Apply Hessian-based Frangi filtering for refinement.

Render video frames using the interactive viewer Napari, based on Python.

REFERENCES AND NOTES

1. H. Sung, J. Ferlay, R. L. Siegel, M. Laversanne, I. Soerjomataram, A. Jemal, F. Bray, Global cancer statistics 2020: Globocan estimates of incidence and mortality worldwide for 36 cancers in 185 countries. *CA Cancer J. Clin.* **71**, 209–249 (2021).

2. B. J. Fueger, P. Clauser, P. Kapetas, N. Pötsch, T. H. Helbich, P. A. T. Baltzer, Can supplementary contrast-enhanced MRI of the breast avoid needle biopsies in suspicious microcalcifications seen on mammography? A systematic review and meta-analysis. *Breast* **56**, 53–60 (2021).

3. H. R. Ferreira Dalla Pria, M. E. Scoggins, T. W. Moseley, V. Vishwa, S. Jean, S. Vuong, V. Diaz, A. Elhatw, Current status of imaging for breast cancer staging. *Curr. Breast Cancer Rep.* **16**, 126–133 (2024).

4. Y. Wang, Y. Li, Y. Song, C. Chen, Z. Wang, L. Li, M. Liu, G. Liu, Y. Xu, Y. Zhou, Q. Sun, S. Shen, Comparison of ultrasound and mammography for early diagnosis of breast cancer among chinese women with suspected breast lesions: A prospective trial. *Thorac. Cancer.* **13**, 3145–3151 (2022).

5. N. Aristokli, I. Polycarpou, S. C. Themistocleous, D. Sophocleous, I. Mamais, Comparison of the diagnostic performance of magnetic resonance imaging (MRI), ultrasound and mammography for detection of breast cancer based on tumor type, breast density and patient's history: A review. *Radiography* **28**, 848–856 (2022).

6. M. Heijblom, J. M. Klaase, F. M. van den Engh, T. G. van Leeuwen, W. Steenbergen, S. Manohar, Imaging tumor vascularization for detection and diagnosis of breast cancer. *Technol. Cancer Res. Treat.* **10**, 607–623 (2011).

7. N. Sharma, M. McMahon, I. Haigh, Y. Chen, B. J. G. Dall, The potential impact of digital breast tomosynthesis on the benign biopsy rate in women recalled within the UK breast screening programme. *Radiology* **291**, 310–317 (2019).

8. R. G. Blanks, R. Given-Wilson, R. Alison, J. Jenkins, M. G. Wallis, An analysis of 11.3 million screening tests examining the association between needle biopsy rates and cancer

detection rates in the English NHS breast cancer screening programme. *Clin. Radiol.* **74**, 384–389 (2019).

9. L. V. Wang, S. Hu, Photoacoustic tomography: In vivo imaging from organelles to organs. *Science* **335**, 1458–1462 (2012).

10. Y. Zhou, J. Yao, L. V. Wang, Tutorial on photoacoustic tomography. *J. Biomed. Opt.* **21**, 061007 (2016).

11. D. Hanahan, R. A. Weinberg, Hallmarks of cancer: The next generation. *Cell* **144**, 646–674 (2011).

12. J. Folkman, Angiogenesis in cancer, vascular, rheumatoid and other disease. *Nat. Med.* **1**, 27–30 (1995).

13. J. E. Bluff, S. R. Menakuru, S. S. Cross, S. E. Higham, S. P. Balasubramanian, N. J. Brown, M. W. Reed, C. A. Staton, Angiogenesis is associated with the onset of hyperplasia in human ductal breast disease. *Br. J. Cancer* **101**, 666–672 (2009).

14. P. M. Carpenter, W. P. Chen, A. Mendez, C. E. McLaren, M. Y. Su, Angiogenesis in the progression of breast ductal proliferations. *Int. J. Surg. Pathol.* **19**, 335–341 (2011).

15. S. C. Heffelfinger, R. Yassin, M. A. Miller, E. Lower, Vascularity of proliferative breast disease and carcinoma in situ correlates with histological features. *Clin. Cancer Res.* **2**, 1873–1878 (1996).

16. N. B. Teo, B. S. Shoker, C. Jarvis, L. Martin, J. P. Sloane, C. Holcombe, Vascular density and phenotype around ductal carcinoma in situ (DCIS) of the breast. *Br. J. Cancer* **86**, 905–911 (2002).

17. N. Weidner, J. P. Semple, W. R. Welch, J. Folkman, Tumor angiogenesis and metastasis - Correlation in invasive breast carcinoma. *N. Engl. J. Med.* **324**, 1–8 (1991).

18. J. Folkman, Role of angiogenesis in tumor growth and metastasis. *Semin. Oncol.* **29**, 15–18 (2002).

19. J. A. Nagy, S. H. Chang, A. M. Dvorak, H. F. Dvorak, Why are tumour blood vessels abnormal and why is it important to know? *Br. J. Cancer* **100**, 865–869 (2009).

20. M. S. Gordon, D. S. Mendelson, G. Kato, Tumor angiogenesis and novel antiangiogenic strategies. *Int. J. Cancer* **126**, 1777–1787 (2010).

21. L. Lin, L. V. Wang, The emerging role of photoacoustic imaging in clinical oncology. *Nat. Rev. Clin. Oncol.* **19**, 365–384 (2022).

22. L. Lin, X. Tong, P. Hu, M. Invernizzi, L. Lai, L. V. Wang, Photoacoustic computed tomography of breast cancer in response to neoadjuvant chemotherapy. *Adv. Sci.* **8**, 2003396 (2021).

23. L. Lin, P. Hu, J. Shi, C. M. Appleton, K. Maslov, L. Li, R. Zhang, L. V. Wang, Single-breath-hold photoacoustic computed tomography of the breast. *Nat. Commun.* **9**, 2352 (2018).

24. L. Lin, P. Hu, X. Tong, S. Na, R. Cao, X. Yuan, D. C. Garrett, J. Shi, K. Maslov, L. V. Wang, High-speed three-dimensional photoacoustic computed tomography for preclinical research and clinical translation. *Nat. Commun.* **12**, 882 (2021).

25. Y. Matsumoto, Y. Asao, H. Sekiguchi, A. Yoshikawa, T. Ishii, K. I. Nagae, S. Kobayashi, I. Tsuge, S. Saito, M. Takada, Y. Ishida, M. Kataoka, T. Sakurai, T. Yagi, K. Kabashima, S. Suzuki, K. Togashi, T. Shiina, M. Toi, Visualising peripheral arterioles and venules through high-resolution and large-area photoacoustic imaging. *Sci. Rep.* **8**, 14930 (2018).

26. I. Yamaga, N. Kawaguchi-Sakita, Y. Asao, Y. Matsumoto, A. Yoshikawa, T. Fukui, M. Takada, M. Kataoka, M. Kawashima, E. Fakhrejahani, S. Kanao, Y. Nakayama, M. Tokiwa, M. Torii, T. Yagi, T. Sakurai, H. Haga, K. Togashi, T. Shiina, M. Toi, Vascular branching point counts using photoacoustic imaging in the superficial layer of the breast: A potential biomarker for breast cancer. *Photoacoustics* **11**, 6–13 (2018).

27. A. Oraevsky, R. Su, H. Nguyen, J. Moore, Y. Lou, S. Bhadra, L. Forte, M. Anastasio, W. Yang, "Full-view 3D imaging system for functional and anatomical screening of the breast,"

in *Proceedings Volume 10494, Photons Plus Ultrasound: Imaging and Sensing 2018*, San Francisco, CA, USA, 11 April 2018 (SPIE, 2018); https://doi.org/10.1117/12.2318802.

28. S. M. Schoustra, B. De Santi, T. J. P. M. Op ‘t Root, C. A. H. Klazen, M. van der Schaaf, J. Veltman, W. Steenbergen, S. Manohar, Imaging breast malignancies with the twente photoacoustic mammoscope 2. *PLOS ONE* **18**, e0281434 (2023).

29. G. Zhang, W. Li, M. Yang, C. Li, Developing a photoacoustic whole-breast imaging system based on the synthetic matrix array. *Front. Phys.* **8**, 600589 (2020).

30. N. Nyayapathi, R. Lim, H. Zhang, W. Zheng, Y. Wang, M. Tiao, K. W. Oh, X. C. Fan, E. Bonaccio, K. Takabe, J. Xia, Dual scan mammoscope (DSM)-A new portable photoacoustic breast imaging system with scanning in craniocaudal plane. *IEEE Trans. Biomed. Eng.* **67**, 1321–1327 (2020).

31. E. Zheng, H. Zhang, S. Goswami, I. E. Kabir, M. M. Doyley, J. Xia, Second-generation dual scan mammoscope with photoacoustic, ultrasound, and elastographic imaging capabilities. *Front. Oncol.* **11**, 779071 (2021).

32. T. Han, M. Yang, F. Yang, L. Zhao, Y. Jiang, C. Li, A three-dimensional modeling method for quantitative photoacoustic breast imaging with handheld probe. *Photoacoustics* **21**, 100222 (2020).

33. H. Zhang, E. Zheng, W. Zheng, C. Huang, Y. Xi, Y. Cheng, S. Yu, S. Chakraborty, E. Bonaccio, K. Takabe, X. C. Fan, W. Xu, J. Xia, OneTouch automated photoacoustic and ultrasound imaging of breast in standing pose. *IEEE Trans. Med. Imaging* (2025).

34. M. Heijblom, D. Piras, F. M. van den Engh, M. van der Schaaf, J. M. Klaase, W. Steenbergen, S. Manohar, The state of the art in breast imaging using the twente photoacoustic mammoscope: Results from 31 measurements on malignancies. *Eur. Radiol.* **26**, 3874–3887 (2016).

35. Y. Asao, Y. Hashizume, T. Suita, K. I. Nagae, K. Fukutani, Y. Sudo, T. Matsushita, S. Kobayashi, M. Tokiwa, I. Yamaga, E. Fakhrejahani, M. Torii, M. Kawashima, M. Takada, S.

Kanao, M. Kataoka, T. Shiina, M. Toi, Photoacoustic mammography capable of simultaneously acquiring photoacoustic and ultrasound images. *J. Biomed. Opt.* **21**, 116009 (2016).

36. X. Tong, C. Z. Liu, Y. Lou, L. Lin, J. Dzubnar, M. Invernizzi, S. D. Santos, Y. Zhang, R. Cao, P. Hu, J. Zheng, J. Torres, A. Kasabyan, L. L. Lai, L. D. Yee, L. V. Wang, Panoramic photoacoustic computed tomography with learning-based classification enhances breast lesion characterization. *Nat. Biomed. Eng.*, 10.1038/s41551-025-01435-3 (2025).

37. M. Dantuma, F. Lucka, S. C. Kruitwagen, A. Javaherian, L. Alink, R. P. Pompe van Meerdervoort, M. Nanninga, T. J. P. M. Po ‘t Root, B. De Santi, J. Budisky, G. Bordovsky, E. Coffy, M. Wilm, T. Kasponas, S. H. Aarnink, L. F. de Geus-Oei, F. Brochin, T. Martinez, A. Michailovas, W. Muller Mobold, J. Jaros, J. Veltman, B. Cox, S. Manohar, Fully three-dimensional sound speed-corrected multi-wavelength photoacoustic breast tomography. arXiv:2308.06754 [physics.med-ph] (2023).

38. G. L. G. Menezes, R. M. Pijnappel, C. Meeuwis, R. Bisschops, J. Veltman, P. T. Lavin, M. J. van de Vijver, R. M. Mann, Downgrading of breast masses suspicious for cancer by using optoacoustic breast imaging. *Radiology* **288**, 355–365 (2018).

39. E. I. Neuschler, R. Butler, C. A. Young, L. D. Barke, M. L. Bertrand, M. Böhm-Vélez, S. Destounis, P. Donlan, S. R. Grobmyer, J. Katzen, K. A. Kist, P. T. Lavin, E. V. Makariou, T. M. Parris, K. J. Schilling, F. L. Tucker, B. E. Dogan, A pivotal study of optoacoustic imaging to diagnose benign and malignant breast masses: A new evaluation tool for radiologists. *Radiology* **287**, 398–412 (2018).

40. B. E. Dogan, G. L. G. Menezes, R. S. Butler, E. I. Neuschler, R. Aitchison, P. T. Lavin, F. L. Tucker, S. R. Grobmyer, P. M. Otto, A. T. Stavros, Optoacoustic imaging and gray-scale US features of breast cancers: Correlation with molecular subtypes. *Radiology* **292**, 564–572 (2019).

41. US Food and Drug Administration, Summary of safety and effectiveness data (ssed): Imagio® Breast Imaging System – P200003 (2021); www.fda.gov/medical-devices/recently-approved-devices/imagior-breast-imaging-system-p200003.

42. M. Yang, L. Zhao, F. Yang, N. Su, C. Zhao, Y. Gui, Y. Wei, R. Zhang, J. Li, T. Han, X. He, L. Zhu, H. Wu, C. Li, Y. Jiang, Quantitative analysis of breast tumours aided by three-dimensional photoacoustic/ultrasound functional imaging. *Sci. Rep.* **10**, 8047 (2020).

43. B. Huang, J. Xia, K. Maslov, L. V. Wang, Improving limited-view photoacoustic tomography with an acoustic reflector. *J. Biomed. Opt.* **18**, 110505 (2013).

44. K. Maslov, G. Stoica, L. V. Wang, In vivo dark-field reflection-mode photoacoustic microscopy. *Opt. Lett.* **30**, 625–627 (2005).

45. S. A. S. Karam, D. O'Loughlin, B. L. Oliveira, M. O'Halloran, B. M. Asl, Weighted delay-and-sum beamformer for breast cancer detection using microwave imaging. *Measurement* **177**, 109283 (2021).

46. S. Hakakzadeh, S. M. Mostafavi, Z. Kavehvash, "Unipolar back-projection algorithm for photoacoustic tomography," in *2022 IEEE International Ultrasonics Symposium (IUS)* (IEEE, 2022), pp. 1–4.

47. S. Tzoumas, A. Nunes, I. Olefir, S. Stangl, P. Symvoulidis, S. Glasl, C. Bayer, G. Multhoff, V. Ntziachristos, Eigenspectra optoacoustic tomography achieves quantitative blood oxygenation imaging deep in tissues. *Nat. Commun.* **7**, 12121 (2016).

48. S. S. S. Choi, B. Lashkari, E. Dovlo, A. Mandelis, Wavelength-modulated differential photoacoustic radar imager (WM-DPARI): Accurate monitoring of absolute hemoglobin oxygen saturation. *Biomed. Opt. Express* **7**, 2586–2596 (2016).

49. T. Zhao, A. E. Desjardins, S. Ourselin, T. Vercauteren, W. Xia, Minimally invasive photoacoustic imaging: Current status and future perspectives. *Photoacoustics* **16**, 100146 (2019).

50. Z. Fang, C. Wang, J. Yang, Z. Song, C. Xie, Y. Ji, Z. Wang, X. Du, Q. Zheng, C. Chen, Z. Hu, Y. Zhong, Oxyhaemoglobin saturation NIR-IIb imaging for assessing cancer metabolism and predicting the response to immunotherapy. *Nat. Nanotechnol.* **19**, 124–130 (2024).

51. Q. Zhu, A. Jr Ricci, P. Hegde, M. Kane, E. Cronin, A. Merkulov, Y. Xu, B. Tavakoli, S. Tannenbaum, Assessment of functional differences in malignant and benign breast lesions and improvement of diagnostic accuracy by using US-guided diffuse optical tomography in conjunction with conventional US. *Radiology* **280**, 387–397 (2016).

52. W. A. Berg, L. Gutierrez, M. S. NessAiver, W. B. Carter, M. Bhargavan, R. S. Lewis, O. B. Ioffe, Diagnostic accuracy of mammography, clinical examination, US, and MR imaging in preoperative assessment of breast cancer. *Radiology* **233**, 830–849 (2004).

53. H. M. Langevin, J. A. Yandow, Relationship of acupuncture points and meridians to connective tissue planes. *Anat. Rec.* **269**, 257–265 (2002).

54. A. C. Ahn, M. Park, J. R. Shaw, C. A. McManus, T. J. Kaptchuk, H. M. Langevin, Electrical impedance of acupuncture meridians: The relevance of subcutaneous collagenous bands. *PLOS ONE* **5**, e11907 (2010).

55. W.-B. Zhang, G.-J. Wang, K. Fuxe, Classic and modern meridian studies: A review of low hydraulic resistance channels along meridians and their relevance for therapeutic effects in traditional chinese medicine. *Evid. Based Complement. Alternat. Med.* **2015**, 410979 (2015).

56. *125Q uestions: Exploration and Discovery* (Science/AAAS Custom Publishing Office, 2021).

57. S. C. Cheng, C. H. Lin, Y. J. Chang, T. H. Lee, S. J. Ryu, C. H. Chen, H. K. Chang, C. J. Chang, W. L. Hu, Y. C. Hung, Fire-heat and Qi deficiency syndromes as predictors of short-term prognosis of acute ischemic stroke. *J. Altern. Complement. Med.* **19**, 721–728 (2013).

58. S. Schröder, J. Liepert, A. Remppis, J. H. Greten, Acupuncture treatment improves nerve conduction in peripheral neuropathy. *Eur. J. Neurol.* **14**, 276–281 (2007).

59. Q. Ji, Y. Q. Luo, W. H. Wang, X. Liu, Q. Li, S. B. Su, Research advances in traditional Chinese medicine syndromes in cancer patients. *J. Integr. Med.* **14**, 12–21 (2016).

60. P. Hu, L. Li, L. V. Wang, Location-dependent spatiotemporal antialiasing in photoacoustic computed tomography. *IEEE Trans. Med. Imaging* **42**, 1210–1224 (2023).

61. “American national standard for the safe use of lasers” (ANSI Z136.1–2022, Laser Institute of America, 2022).

62. L. V. Wang, H. I. Wu, *Biomedical Optics: Principles and Imaging* (John Wiley & Sons Press, 2007).

63. R. G. Barr, A. Rim, R. Graham, W. Berg, J. R. Grajo, Speed of sound imaging improved image quality in breast sonography. *Ultrasound Q.* **25**, 141–144 (2009).

64. J. A. Jensen, S. I. Nikolov, K. L. Gammelmark, M. H. Pedersen, Synthetic aperture ultrasound imaging. *Ultrasonics* **44**, E5–E15 (2006).

65. V. Perrot, M. Polichetti, F. Varray, D. Garcia, So you think you can DAS? A viewpoint on delay-and-sum beamforming. *Ultrasonics* **111**, 106309 (2021).

66. S. Arridge, P. Beard, M. Betcke, B. Cox, N. Huynh, F. Lucka, O. Ogunlade, E. Zhang, Accelerated high-resolution photoacoustic tomography via compressed sensing. *Phys. Med. Biol.* **61**, 8908–8940 (2016).

67. K. B. Chowdhury, J. Prakash, A. Karlas, D. Justel, V. Ntziachristos, A synthetic total impulse response characterization method for correction of hand-held optoacoustic images. *IEEE Trans. Med. Imaging* **39**, 3218–3230 (2020).

68. N. Awasthi, G. Jain, S. K. Kalva, M. Pramanik, P. K. Yalavarthy, Deep neural network-based sinogram super-resolution and bandwidth enhancement for limited-data photoacoustic tomography. *IEEE Trans. Ultrason. Ferroelectr. Freq. Control* **67**, 2660–2673 (2020).

69. H. Shan, G. Wang, Y. Yang, Accelerated correction of reflection artifacts by deep neural networks in photo-acoustic tomography. *Appl. Sci.* **9**, 2615 (2019).

70. N. Davoudi, X. L. Dean-Ben, D. Razansky, Deep learning optoacoustic tomography with sparse data. *Nat. Mach. Intell.* **1**, 453–460 (2019).

71. L. Li, L. Zhu, C. Ma, L. Lin, J. Yao, L. Wang, K. Maslov, R. Zhang, W. Chen, J. Shi, L. V. Wang, Single-impulse panoramic photoacoustic computed tomography of small-animal whole-body dynamics at high spatiotemporal resolution. *Nat. Biomed. Eng.* **1**, 0071 (2017).

72. K. Zuiderveld, "Contrast limited adaptive histogram equalization," in *Graphics Gems IV* (Academic Press Professional Inc., 1994), pp. 474–485.

73. J. Kim, J. K. Lee, K. M. Lee, "Accurate image super-resolution using very deep convolutional networks," in *2016 IEEE Conference on Computer Vision and Pattern Recognition (CVPR)* (IEEE, 2016), pp. 1646–1654.

74. S. Ghavami, M. Bayat, M. Fatemi, A. Alizad, Quantification of morphological features in non-contrast-enhanced ultrasound microvasculature imaging. *IEEE Access* **8**, 18925–18937 (2020).

75. R. Ternifi, Y. Wang, J. Gu, E. C. Polley, J. M. Carter, S. Pruthi, J. C. Boughey, R. T. Fazzio, M. Fatemi, A. Alizad, Ultrasound high-definition microvasculature imaging with novel quantitative biomarkers improves breast cancer detection accuracy. *Eur. Radiol.* **32**, 7448–7462 (2022).

76. *BI-RADS®: ACR Breast Imaging Reporting and Data System* (American College of Radiology, 2013).

77. W. Gomez-Flores, J. Hernandez-Lopez, Assessment of the invariance and discriminant power of morphological features under geometric transformations for breast tumor classification. *Comput. Methods Programs Biomed.* **185**, 105173 (2020).

78. B. Karimi, A. Krzyzak, A novel technique for detecting suspicious lesions in breast ultrasound images. *Concurr. Comput. Pract. Exper.* **28**, 2237–2260 (2016).

79. S. Raza, A. L. Goldkamp, S. A. Chikarmane, R. L. Birdwell, US of breast masses categorized as BI-RADS 3, 4, and 5: Pictorial review of factors influencing clinical management. *Radiographics* **30**, 1199–1213 (2010).

80. A. Gronningsaeter, B. A. J. Angelsen, A. Heimdal, H. G. Torp, Vessel wall detection and blood noise reduction in intravascular ultrasound imaging. *IEEE Trans. Ultrason. Ferroelectr. Freq. Control* **43**, 359–369 (1996).

81. A. Altmann, L. Tolosi, O. Sander, T. Lengauer, Permutation importance: A corrected feature importance measure. *Bioinformatics* **26**, 1340–1347 (2010).

82. "Near-infrared window in biological tissue"; https://en.wikipedia.org/wiki/Near-infrared_window_in_biological_tissue#cite_note-10.

83. J. G. Koelzer, G. Mitic, J. Otto, W. Zinth, "Measurements of the optical properties of breast tissue using time-resolved transillumination," in *Proceedings Volume 2326, Photon Transport in Highly Scattering Tissue*, Lille, France, 31 January 1995 (SPIE, 1995); https://doi.org/10.1117/12.200841.

84. S. A. Ermilov, T. Khamapirad, A. Conjusteau, M. H. Leonard, R. Lacewell, K. Mehta, T. Miller, A. A. Oraevsky, Laser optoacoustic imaging system for detection of breast cancer. *J. Biomed. Opt.* **14**, 024007 (2009).

85. D. Garcia, "Make the most of MUST, an open-source Matlab Ultrasound Toolbox," *2021 IEEE International Ultrasonics Symposium (IUS)* (IEEE, 2021), pp. 1–4.

86. D. D. Royston, R. S. Poston, S. A. Prahl, Optical properties of scattering and absorbing materials used in the development of optical phantoms at 1064 nm. *J. Biomed. Opt.* **1**, 110–116 (1996).

87. R. Michels, F. Foschum, A. Kienle, Optical properties of fat emulsions. *Opt. Express* **16**, 5907–5925 (2008).

88. T. Hopp, A. Stromboni, N. Duric, N. V. Ruiter, "Evaluation of breast tissue characterization by ultrasound computer tomography using a 2D/3D image registration with mammograms," in *2013 IEEE International Ultrasonics Symposium (IUS)* (IEEE, 2013), pp. 647–650.

89. A. Jozefczak, K. Kaczmarek, M. Kubovcikova, Z. Rozynek, T. Hornowski, The effect of magnetic nanoparticles on the acoustic properties of tissue-mimicking agar-gel phantoms. *J. Magn. Magn. Mater.* **431**, 172–175 (2017).
